# Molecular and spatial specialization of lung interstitial macrophage subsets: beyond chemokines

**DOI:** 10.3389/fimmu.2026.1824159

**Published:** 2026-06-10

**Authors:** Xin Li, Claudia V. Jakubzick

**Affiliations:** Department of Microbiology and Immunology, Dartmouth Geisel School of Medicine, Hanover, NH, United States

**Keywords:** lung, interstitial macrophage, spatial transcriptomics, macrophage heterogeneity, chemokine, cytokine, complement, innate immunity

## Abstract

**Introduction:**

Interstitial macrophages (IMs) are increasingly recognized for their vital roles in maintaining tissue homeostasis and orchestrating immune responses. Building on earlier work showing that two overarching IM subsets, CD206hi and CD206lo, encompass ten unique chemokine-expressing subpopulations that regulate immune cell recruitment and tertiary lymphoid structures, we sought to further define the molecular programs, potential divisions of labor, and spatial organization of murine lung IMs.

**Methods:**

We performed a comprehensive transcriptomic analysis of murine lung IMs and integrated these data with Xenium spatial transcriptomics to examine IM subset-associated gene programs and localization within the lung microenvironment. Differential gene expression across IM subsets is summarized in accompanying tables.

**Results:**

CD206hi and CD206lo IM subsets exhibited distinct cytokine and receptor gene profiles, along with a predicted autocrine network that may influence their migration and cytokine-driven functions. IM subsets also displayed distinct innate immune signatures, including complement components, scavenger receptors, and pattern recognition receptors, such as Toll-like receptors and C-type lectins. Using Xenium spatial transcriptomics, we found that IMs in our dataset predominantly localized to three lung regions: bronchovascular bundles, interstitium, and periphery. CD206hi and CD206lo IMs preferentially occupied specific anatomical niches, associated with differential integrin and metallopeptidase gene expression. Chemokine expression within IMs also showed distinct spatial localization patterns associated with the positioning of T cells and B cells.

**Discussion:**

Overall, our findings advance the understanding of IM heterogeneity and identify molecular programs associated with chemoattraction, inflammation regulation, innate immune defense, and tissue maintenance, while providing a high-resolution framework for investigating their localization, interactions, and contributions to lung immunity and disease.

## Introduction

Macrophages are versatile immune cells that maintain tissue homeostasis and serve as first-line defenders against immunological challenges. In the lung, two types of tissue-resident macrophages exist: alveolar macrophages (AMs), which occupy the airspaces and are unique to the lung, and interstitial macrophages (IMs), which reside within the connective tissue/interstitial compartment rather than the epithelial surface. Unlike highly tissue-specialized macrophages such as AMs, IMs represent a broadly conserved tissue-resident macrophage population found across multiple organs, where they occupy stromal, perivascular, peribronchial, and nerve-associated niches and perform more general interstitial functions related to immune surveillance, tissue support, and local immune regulation (1–8). While some IMs are found in the airspace, they remain distinct from AMs in their location, transcriptional profiles, and function (2, 9–12). A separate population of macrophages, often referred to as surveying, trafficking, or recruited monocytes/macrophages is present at steady state and dramatically increases during inflammation (13–15). Recruited macrophages can differentiate into antigen-presenting, pro- or anti-inflammatory macrophages but do not establish long-term residence unless replacing the tissue-resident macrophage niche (14). This study focuses on tissue-resident IMs, and not the recruited macrophages.

IMs are increasingly implicated in lung diseases characterized by chronic inflammation, tissue remodeling, infection, allergy, fibrosis, and tumor-associated immune regulation (1, 4, 16–21). Prior studies have shown that IMs can regulate dendritic-cell function during airway allergy, restrain neutrophilic asthma-like inflammation through IL-10, contribute to fibrotic lung remodeling, maintain immune homeostasis during bacterial dysbiosis-associated inflammation, and support lung tumor growth in an IL-9-dependent model (1, 16–20). Because IMs occupy interstitial, perivascular, peribronchial, and nerve-associated niches, they are well positioned to influence leukocyte recruitment, antigen presentation, extracellular matrix remodeling, and local inflammatory tone (3, 8, 22, 23). Therefore, mapping the molecular and spatial organization of IM subsets in healthy and inflamed lungs provides an important framework for understanding how these cells may contribute to disease pathology.

To define these tissue-resident IM populations more precisely, prior studies have used flow cytometry to distinguish IMs from AMs based on the expression of Siglec-F, CD11c, and CD11b, with IMs further classified into two subsets. One subset expresses CD11c, CCR2, CX_3_CR1, and MHCII, while the other, which has higher CD206 expression, also expresses CD163, FRβ (*Folr2*), and varying levels of MHCII, CX_3_CR1, and LYVE1 (22). Our lab and others have examined, identified, and demonstrated these bona fide gene expression patterns in pulmonary CD206^hi^ and CD206^lo^ IM subsets via bulk-RNA sequencing and single-cell RNA sequencing (scRNA-seq) (3, 22–24).

Pathway analysis of transcriptome data suggests that CD206^hi^ IMs have enhanced phagocytic capacity, while CD206^lo^ IMs are more specialized for antigen presentation, aligning with their respective surface marker profiles (3, 22, 23). However, the specific roles of these subsets remain to be fully elucidated, as IM-subset-specific depletion models are lacking or have various limitations (3, 5, 25). Recently, our group identified at least ten distinct IM subsets, each with specialized chemokine expression patterns, indicating a division of labor for recruiting immune cells across different contexts (3). Selective depletion of CD206^hi^ IMs eliminates IM subsets that produce key chemokines (*Cxcl13, Cxcl9, and Cxcl10*) essential for recruiting B cells and T cells, which disrupts the formation of inducible bronchus-associated lymphoid tissues (iBALTs) (3, 21), underlining the importance of these chemokine-driven specializations.

Here, we extend IM subset analysis beyond chemokines in both naive lungs and lungs exposed to intranasal lipopolysaccharide (LPS), a commonly used experimental model of acute lung injury (ALI) that captures key inflammatory features of acute respiratory distress syndrome (ARDS) (26–28). We uncover additional heterogeneous expression patterns in cytokine and receptor genes, while identifying potential autocrine signaling networks. We also highlight a division of labor within innate immune pathways involving complement components, scavenger receptors, and pattern recognition receptors. Furthermore, spatial transcriptomics via Xenium reveals that CD206^hi^ and CD206^lo^ IMs and their chemokine-expressing subsets occupy distinct lung niches. Together, our findings delineate a complex molecular and spatial landscape of IM subsets and provide a framework for future functional studies.

## Results

### Overview of differential gene expression across IM subsets

First, we provide two tables summarizing some of the most significant analytical data presented in this manuscript. **Table 1** highlights differences in gene expression between CD206^hi^ and CD206^lo^ IM subsets in both stimulated and unstimulated conditions. **Table 2** compares gene expression among IMck subsets (IMck1–9), emphasizing their distinct chemokine signatures and expression profiles across various functional systems.

**Table 1:** Summarized differential gene expression between the CD206^hi^ and CD206^lo^ IM subsets.

Gene category	By subsets (both LPS and naive)		By stimulation (both subsets)		By subsets and stimulation			
	CD206^hi^	CD206^lo^	LPS	Naive	CD206^hi^_LPS	CD206^hi^_Naive	CD206^lo^_LPS	CD206^lo^_Naive
Chemokine Receptors				*Cx3cr1*, *Cxcr4*			*Ccr5*	*Ccr2*, *Ccrl2*
Cytokines	*Kitl*, *Il10*, *Grn*	*Il1b*, *Tnf*, *Tnfsf13b*, *Osm*	*Il1rn*		*Timp1*	*Tslp*, *Bmp2*, *Kitl*		*Tnf*
Cytokine Receptors	*Il6st*, *Il21r*, *Tnfrsf1a*, *Csf3r*	*Cd74*, *Il7r*, *Cd44*, *Tnfrsf12a*, *Il18rap*	*Gpr35*, *Il1rap*	*Il10ra*, *Ifngr1*, *Il1rl1*, *Il13ra1*, *Il10rb*	*Il4ra*, *Gfra4*, *Il2rg*	*Il6ra*, *Gfra2*, *Epor*, *Lifr*, *Tnfrsf17*, *Tnfrsf25*, *Cd4*		*Il18r1*
Complement	*Cfp*, *Cd55*, *C3*	*Itgax*, *Il1b*	*C2*, *C1qbp*	*Ighm*	*Cd59a*	*Cfh*, *C4b*, *Fcna*, *Vsig4*, *C6*		
Scavenger Receptors	*Stab1*, *Cd163*, *Colec12*		*Msr1*		*Enpp1*, *Scarf1*, *Enpp2*			
PRRs	*Ninj1*	*Scimp*, *Traf3ip3*, *Havcr2*	*Tlr1*, *Ecsit*, *Tnip3*, *Clec4n*, *Clec4d*, *Nr1h3*, *Usp50*, *Phb2*	*Unc93b1*, *Tifa*, *Nfkbia*, *Tnfaip3*		*Tlr4*, *Tlr5*, *Tlr7*, *Tlr8*, *Lamp2*, *Slc15a2*, *Ptafr*, *Sec14l1*		
Integrins	*Pmp22*, *Vcam1*, *Ptk2*, *Tln2*, *Egfr*, *Dab2*, *Timp2*, *P2ry12*, *Lamb2*	*Cd9*, *Itgax*, *Itgal*, *F11r*, *Cd81*, *Emilin1*	*Fn1*, *Itga5*, *Actn1*, *Adam8*	*Tln1*, *Itga4*, *Itgb5*, *Tspan8*				
MMPs	*Mmp9*, *Adam9*, *Adam33*, *Bmp1*, *Pepd*	*Mmp12*, *Mmp13*, *Amz1*, *Dpep2*, *Adamdec1*	*Anpep*, *Mmp14*, *Cpd*, *Mmp19*, *Ece2*, *Thop1*, *Psmd7*, *Lap3*, *Xpnpep1*	*Adam19*, *Enpep*, *Cpq*				

IMs universally express certain chemokine and complement genes, including *Pf4*, *Cxcl16*, *Cklf*, C1q genes, *C3ar1*, *C5ar1*, *Itgam*, and *Cr1l*.

**Table 2:** Summarized differential gene expression among IMck subsets.

Gene category	IMck1	IMck2	IMck3	IMck4	IMck5	IMck6	IMck7	IMck8	IMck9
Chemokines	*Ccl12*, *Ccl7*, *Ccl2*	*Ccl5*	*Ccl3*, *Ccl4*, *Cxcl1*, *Cxcl2*	*Ccl5*, *Ccl3*, *Ccl4*, *Cxcl1*, *Cxcl2*, *Cxcl3*	*Ccl8*	*Ccl6*, *Ccl9*	*Cxcl10*, *Cxcl9*	*Cxcl13*	*Ccl24*
Cytokines			*Il1b*, *Tnf*, *Osm*	*Il1b*, *Tnf*, *Il6*, *Inhba*, *Osm*, *Csf1*, *Csf3*, *Il1a*, *Gdf15*			*Tnfsf10*, *Il15*, *Csf1*, *Il27*, *Sectm1a*, *Il18*, *Nampt*		*Tslp*, *Bmp2*, *Kitl*
Cytokine Receptors				*Il7r*, *Tnfrsf1b*			*Il15ra*		*Il6ra*, *Gfra2*, *Epor*, *Lifr*, *Tnfrsf17*, *Tnfrsf25*, *Cd4*
Complement				*Rgcc*					*Cfh*, *C4b*, *Fcna*, *Vsig4*, *C6*
PRRs				*Clec4e*, *Cav1*			*Tlr3*, *Trim30a*, *Ddx60*, *Eif2ak2*, *Ifi204*, *Ifih1*, *Dhx58*, *Irf7*		

IMck0 was not included as it does not specifically express genes in those categories, apart from these universally expressed chemokine and complement genes. The PRR-related genes specifically expressed by IMck7 also include: *Pik3ap1*, *Gbp2*, *Gbp5*, *Ifi35*, *Ifi203*, *Ifi205*, *Igtp*, *Irgm1*, *Irgm2*, *Mndal*, *Nmi*, *Oas1g*, *Oas3*, *Oasl1*, *Rsad2*, *Trim12a*, *Trim12c*, *Trim30b*, *Trim30c*, and *Trim30d*.

### Established IM heterogeneity and coordinated chemokine expression

**Figure 1:**
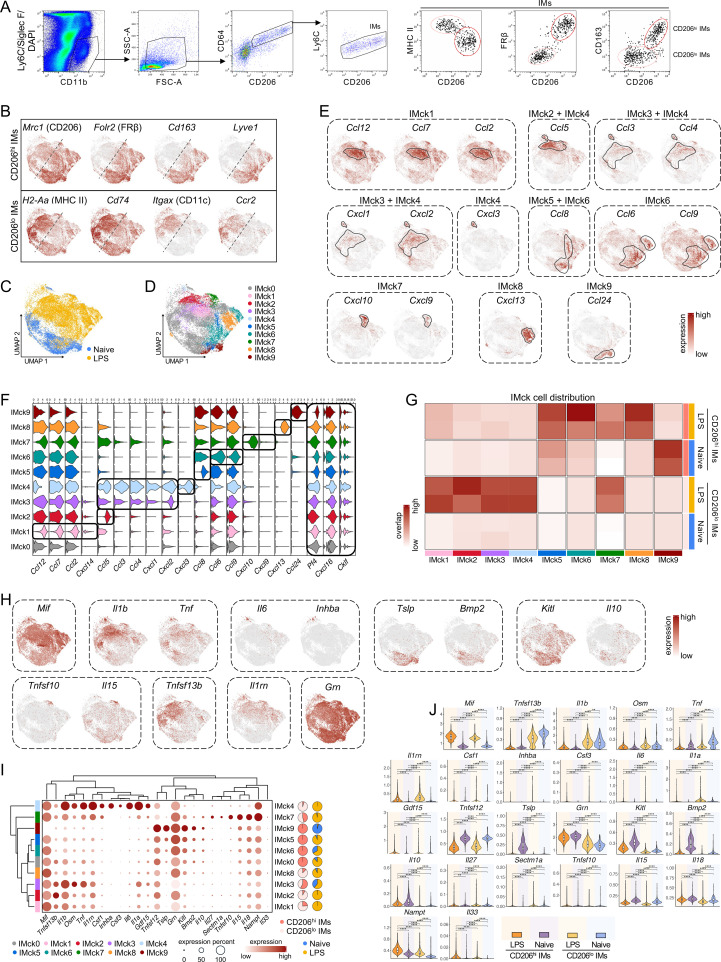
Established IM heterogeneity and coordinated chemokine expression. **(A)** Flow plots demonstrating IM heterogeneity in protein expression between CD206^hi^ and CD206^lo^ IMs. **(B)** Feature plots displaying expression of *Mrc1* (CD206), *Folr2* (FRβ), *Cd163*, *Lyve1*, *H2-Aa* (MHC II), *Cd74*, *Itgax* (CD11c), and *Ccr2* marker genes for CD206^hi^ IMs and CD206^lo^ IMs. **(C)** UMAP plots illustrating the distribution of IM from steady state (Naive) and after 24 h *in vivo* stimulation with LPS. **(D)** UMAP plots illustrating the distribution of IMck0–IMck9 subset. **(E)** Feature plots displaying the differential expression of chemokine genes. The corresponding IMck subsets are circled with a single contour line. **(F)** Violin plot comparing chemokine gene expression across the IMck subsets. **(F)** Heat map visualizing IMck overrepresentation within CD206^hi^/CD206^lo^ IMs under different treatments. **(H)** Feature plots displaying the differential expression of cytokine genes. **(I)** Dot plot highlighting cytokine gene expression across the IMck subsets. Pie charts for each row indicate the IMck overrepresentation within CD206^hi^/CD206^lo^ IMs and IMs under different treatments. **(J)** Violin plot comparing cytokine gene expression across CD206^hi^/CD206^lo^ IMs under different treatments. Within each violin, a box plot spans the interquartile range (25th to 75th percentiles) with a horizontal line at the median; whiskers extend to 1.5 × the interquartile range. P values calculated using Wilcox test. *P < 0.05; **P < 0.01; ***P < 0.001; ****P < 0.0001; nonsignificant results not shown.

First, revisiting previously reported IM classifications, CD206^hi^ and CD206^lo^ IMs exhibit distinct gene expression patterns, consistent with earlier observations from flow cytometry and bulk RNA-seq (**Figures 1A–C**; **Supplementary Figure S1A–B**; **Supplementary Table 1**). Specifically, CD206^hi^ IMs are *Folr2*^+^ *Cd163*^+^ *Lyve1*^+/-^ *H2-Aa*^lo^ *Cd74*^lo^ *Itgax*^-^ *Ccr2*^-^ (or FRβ^+^CD163^+^LYVE1^+^MHC II^-/+^CD74^-/lo^CD11c^-^CCR2^-^ for protein), whereas CD206^lo^ IMs are *Folr2*^-^ *Cd163*^-^ *Lyve1*^-^ *H2-Aa*^hi^ *Cd74*^hi^ *Itgax*^+^ *Ccr2*^+^ (or FRβ^-^CD163^-^LYVE1^-^MHC II^+^CD74^+^CD11c^+^CCR2^+^ for protein) (22). Beyond these two subsets, our earlier study proposed an alternative chemokine-based classification of interstitial macrophages, termed IMck subsets (interstitial macrophage chemokine subsets; IMck0–9), based on coordinated chemokine gene expression, analogous to the division of CD4^+^ T cells into Th1, Th2, Th17, and Treg subsets (3). In this nomenclature, “ck” refers to chemokine expression, and each IMck subset is defined by a characteristic “core” set of chemokine signature genes while also sharing several common chemokines (e.g., *Pf4* [CXCL4], *Cxcl16*, *Cklf*) (**Figures 1D–F**; **Supplementary Figure S1C–D**; **Supplementary Table 2**). In this figure and subsequent figures, dotted boxes indicate similar gene expression patterns. An interactive version of the dataset is available at https://cells.ucsc.edu/?ds=macrophage-atlas+lung-im (29).

It is important to note that IMck subsets are distributed across both CD206^hi^ and CD206^lo^ IM populations, with some subsets being predominantly represented by one group (**Figure 1G**). For example, the *Cxcl13*-expressing IMck8 is enriched in CD206^hi^ IMs; whereas the *Cxcl3*-expressing IMck4 (previously classified as non-classical IMs (3)) is predominantly found in CD206^lo^ IMs. Additionally, while most IMck subsets are enriched following LPS stimulation, the *Ccl24*-expressing IMck9 is particularly abundant in naive IMs.

### Cytokine gene expression among IMs

In addition to chemokines, various cytokine genes exhibit differential expression across IM subsets (**Figures 1H–J**, **S2A–B**). For instance, *Mif* (macrophage migration inhibitory factor) is expressed by all IMck subsets except IMck9 (**Figure 1I**), with the highest levels observed following LPS stimulation (**Figure 1J**). Pro-inflammatory cytokine genes *Il1b* and *Tnf* are predominantly expressed in CD206^lo^ subsets (or IMck3 and IMck4). Additionally, *Il6* and *Inhba* (Activin A) are both enriched in IMck4, indicating a role in acute inflammatory responses. In contrast, *Tslp* and *Bmp2* (a TGF-β family member) are preferentially expressed in naive CD206^hi^ IMs (or IMck9), which also produce *Ccl24* (eotaxin-2), suggesting possible involvement in type 2 immunity (**Figures 1E, F**) (3, 25). Additionally, CD206^hi^ IMs exhibit higher expression of *Kitl* (stem cell factor) and *Il10*, suggesting an immunoregulatory function.

*Tnfsf10*, encoding the apoptosis-inducing TRAIL protein (30), and *Il15*, encoding a key modulator for NK cells and CD8^+^ T cells (31), are both specifically expressed in IMck7, suggesting a potential link to cytotoxic functionality in this interferon-responsive, *Cxcl9*/*Cxcl10*-expressing subset (**Figures 1D–-F, H-J**). Markedly, *Tnfsf13b* (BAFF) is largely expressed by CD206^lo^ IMs, suggesting a potential additional IM–B cell interaction beyond the previously documented CXCL13-CXCR5 axis (3). Moreover, LPS-treated IMs upregulate *Il1rn* (IL-1 receptor antagonist), consistent with a potential negative feedback mechanism to restrain excessive IL-1–mediated inflammation. Altogether, these findings highlight the extensive cytokine repertoire of IMs and how different subsets may be equipped to respond to and potentially shape a wide range of immune contexts (**Figures 1H–J**).

Together, these data suggest that IM subsets differ not only in chemokine expression but also in cytokine programs that may support distinct inflammatory, regulatory, and tissue-associated functions.

### Cytokine and chemokine receptor gene expression among IMs

**Figure 2:**
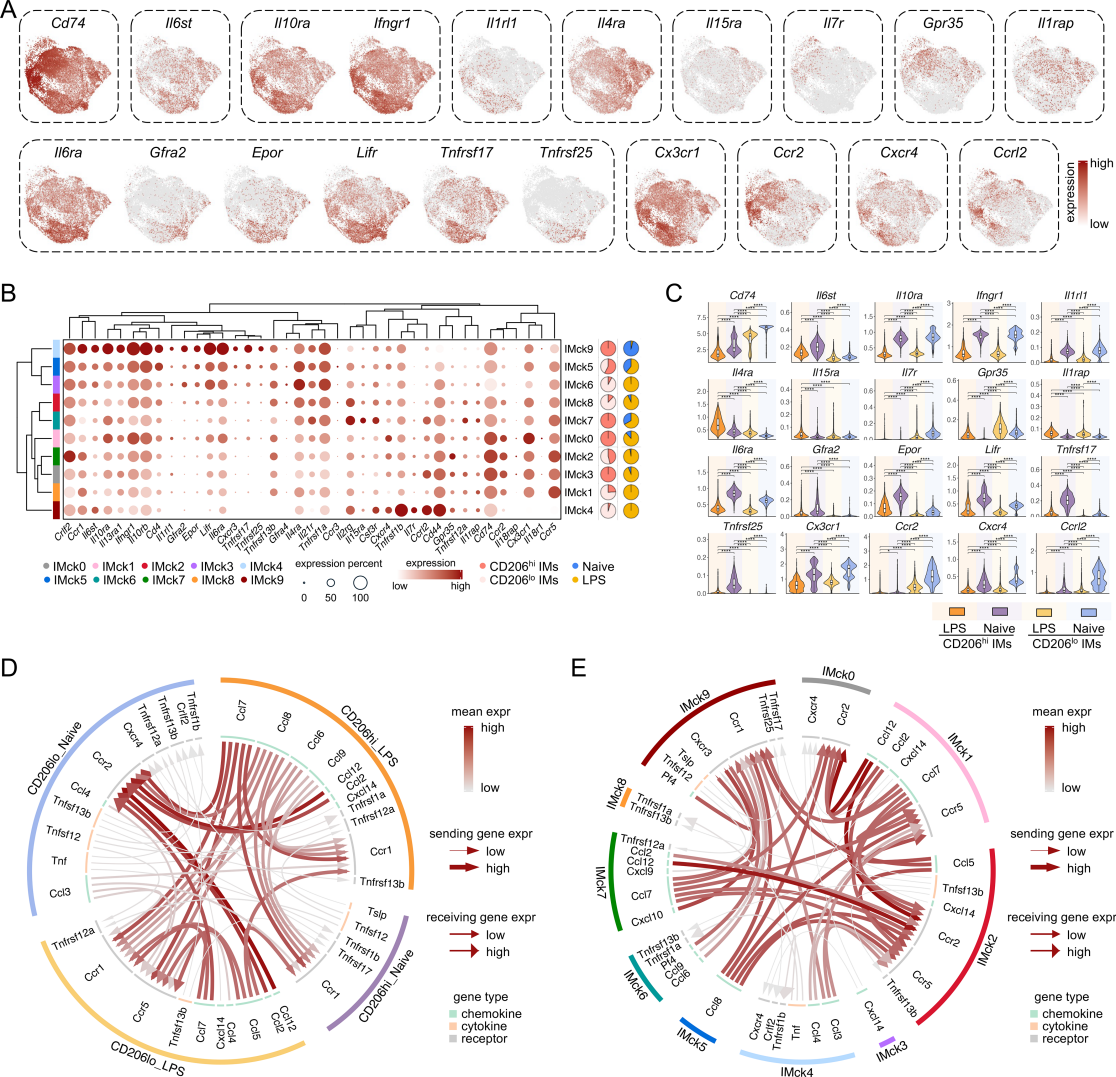
Cytokine and chemokine receptor genes expression among IMs. **(A)** Feature plots displaying the differential expression of cytokine and chemokine receptor genes. **(B)** Dot plot highlighting cytokine and chemokine receptor gene expression across the IMck subsets. Pie charts for each row indicate the IMck overrepresentation within CD206^hi^/CD206^lo^ IMs and IMs under different treatments. **(C)** Violin plot comparing cytokine and chemokine receptor gene expression across CD206^hi^/CD206^lo^ IMs under different treatments. Within each violin, a box plot spans the interquartile range (25th to 75th percentiles) with a horizontal line at the median; whiskers extend to 1.5 × the interquartile range. P values calculated using Wilcox test. *P < 0.05; **P < 0.01; ***P < 0.001; ****P < 0.0001; nonsignificant results not shown. **(D)** Circos plot depicting the top 50 interactions where cytokines and chemokines work in an autocrine manner to direct function of CD206^hi^/CD206^lo^ IMs under different treatments. **(E)** Circos plot depicting the top 50 interactions where cytokines and chemokines work in an autocrine manner to direct function of each IMck subset. Each segment of the outer circle represents a distinct IMck subset. Each segment of the inner circle represents different genes, color-coded by molecule type. Arcs indicate ligand-receptor interactions, with line thickness proportional to the expression of ligand genes, and arrow width proportional to the expression of receptor genes. Arcs are color-coded by mean expression value. CellPhoneDB interactions represent predicted ligand-receptor pairs inferred from gene-expression data and should not be interpreted as direct evidence of active signaling. And “autocrine” here refers to predicted interactions at the overall IM population level; subset-level paracrine interactions also contribute.

Many cytokines produced by IM subsets can act on macrophages themselves, suggesting potential autocrine signaling loops (in this study, autocrine refers to signaling at the overall IM population level, while acknowledging that paracrine effects could occur at the subset level). To investigate this further, we analyzed the differential expression of cytokine and chemokine receptor genes (**Figures 2A–C**; **Supplementary Figures 2C, D**). *Cd74*, which encodes a multifunctional MHC II chaperone, is highly expressed by CD206^lo^ IMs with its protein serving also as a receptor for MIF to activate AKT signaling (32). Meanwhile, *Il6st* (gp130) and *Il6ra* are mainly found in CD206^hi^ IMs, suggesting potential interactions with IL-6 produced by IMck4.

Naive IMs exhibit high levels of *Il10ra* and *Il10rb* (**Figures 2A–C**); however, this does not necessarily indicate a suppressive phenotype, as they also co-express *Ifngr1*, the gene encoding the receptor for the pro-inflammatory cytokine IFN-γ. Rather, naive IMs appear to maintain a diverse receptor profile, allowing them to respond to a broad range of stimuli. Likewise, they also express *Il1rl1* (ST2), a receptor for IL-33, which is primarily released by stromal cells during type 2 immunity (33), likely contributing to reparative or anti-inflammatory responses. While naive IMs express many type 2–associated genes, stimulated CD206^hi^ IMs exhibit higher *Il4ra* expression. However, IL-4’s function in macrophages is complex—although typically associated with type 2 immunity, it can also regulate macrophage residency, DNA repair, and proliferation (34–37). Also noteworthy, IMck7 cells co-express *Il15* and *Il15ra* (**Figures 1H–J**, **2A, B**), suggesting the possibility of a strong autocrine IL-15 loop or the trans-presentation of IL-15 to adjacent lymphocytes (38). Additionally, some CD206^lo^ IMs express *Il7r*, a receptor not typically associated with macrophages.

Under LPS stimulation, various cytokine receptor genes are induced (**Figures 2A–C**), including *Gpr35*, which encodes a G protein–coupled receptor intersecting with MAPK and NF-κB pathways (39). *Il1rap*, encoding the accessory protein for the IL-1 receptor (40), is also among the induced genes, potentially enabling LPS-stimulated IMs to respond to IL-1a and IL-1b produced by CD206^lo^ IMs (or IMck4). Beyond the aforementioned *Il6ra*, naive CD206^hi^ IMs (or the Ccl24-expressing IMck9) highly express *Gfra2*, *Epor*, *Lifr*, *Tnfrsf17*, and *Tnfrsf25*: Epor has been linked to immune tolerance in macrophages (41, 42); *Tnfrsf17* (BCMA) is less studied in macrophages but is suggested to modulate microglial function through the ligands, APRIL and BAFF (43). *Tnfrsf25* encodes a receptor for TNFSF12, which can trigger apoptosis and NF-κB signaling (44, 45).

In addition to these cytokine receptors, IMs express various chemokine receptors, some of which serve as classic markers for distinguishing IM subsets (**Figures 2A–C**, **S2D**). For instance, *Cx3cr1* (CX_3_CR1), a hallmark receptor of monocytes and some tissue-resident macrophages, is predominantly expressed in naive IMs, while *Ccr2* (CCR2) commonly associated with tissue-trafficking monocytes and CD206^lo^ IMs (**Figure 1B**), similarly declines after activation. Additionally, *Cxcr4* (CXCR4, the receptor for CXCL12) is primarily expressed in naive IMs (or IMck4 and IMck0), whereas the atypical receptor *Ccrl2* (CCRL2, the receptor for chemerin) is enriched in IMck3, IMck4, and IMck7.

Thus, IM subsets express distinct receptor programs that may allow them to respond differently to inflammatory, homeostatic, and tissue-derived signals.

### Predicted cytokine and chemokine interactions among IM subsets

To identify candidate cytokine and chemokine interactions among IM subsets, we performed an interactome analysis using CellPhoneDB, a ligand-receptor analysis tool that infers potential cell-cell communication from gene-expression patterns (46) (**Figures 2D, E**; **Supplementary Figure S2E–F**). Accordingly, the interactions identified here are best viewed as candidate communication pathways rather than direct evidence of active signaling. In this study, the term “autocrine” refers to predicted interactions at the overall IM population level, while acknowledging that subset-level paracrine interactions may also occur among distinct IM populations. Most of the identified interactions involved chemokines and their receptors, whereas non-chemoattractant cytokine interactions were fewer and less significant (**Figures 2D, E**). A notable finding was the intricate network involving *Ccr1*, *Ccr2*, and *Ccr5* in both CD206^hi^/CD206^lo^-based IM subsets (**Figure 2D**) and chemokine-based IMcks (**Figure 2E**). These chemokine receptors play a critical role in monocyte/macrophage migration, suggesting that IM subsets may leverage chemokine gradients to reposition themselves under both steady-state and pathological settings. For example, naive CD206^lo^ IMs can be recruited by LPS-activated IMs via CCR2, potentially positioning them to confront threats and initiate adaptive responses, aligning with the antigen-presenting role of CD206^lo^ IMs (3).

Among the fewer non-chemoattractant cytokine–receptor interactions, TNF superfamily members were prominent (**Figures 2D, E**). For instance, The predicted BAFF–BCMA interaction links BAFF (*Tnfsf13b*)-expressing CD206^lo^ IMs with BCMA (*Tnfrsf17*)-expressing naive CD206^hi^ IMs. TNFRSF13B also receives signals from BAFF, exerting autocrine effects in naive CD206^lo^ IMs or establishing a connection between IMck2 and the B-cell–chemoattracting, CXCL13-expressing IMck8 (3). The pro-apoptotic and pro-inflammatory cytokine TNFSF12 may function in an autocrine manner in naive CD206^lo^ IMs via TNFRSF12A (47) and in IMck9 via TNFRSF25 (44, 45). Meanwhile, TNF (*Tnf*) and its receptors TNFRSF1A and TNFRSF1B mediate interactions between naive CD206^lo^ IMs and CD206^hi^ IMs (or IMck4 and IMck6), while also supporting strong autocrine loops in pro-inflammatory IMck4. Another key cytokine is TSLP, primarily enriched in the type 2–oriented IMck9 cluster. Most IMs broadly express *Crlf2* (the TSLP receptor) (**Supplementary Figure 2**), implying that *Tslp*-expressing IMck9 may exert wide regulatory influence among IMs. Altogether, these observations suggest a sophisticated web of candidate cytokine and chemokine signals that shape interactions not only among IM subsets but also potentially with other cell types.

Overall, this analysis identifies candidate cytokine and chemokine interaction networks among IM subsets, while providing hypotheses for future functional testing.

### Complement gene expression among IMs

**Figure 3:**
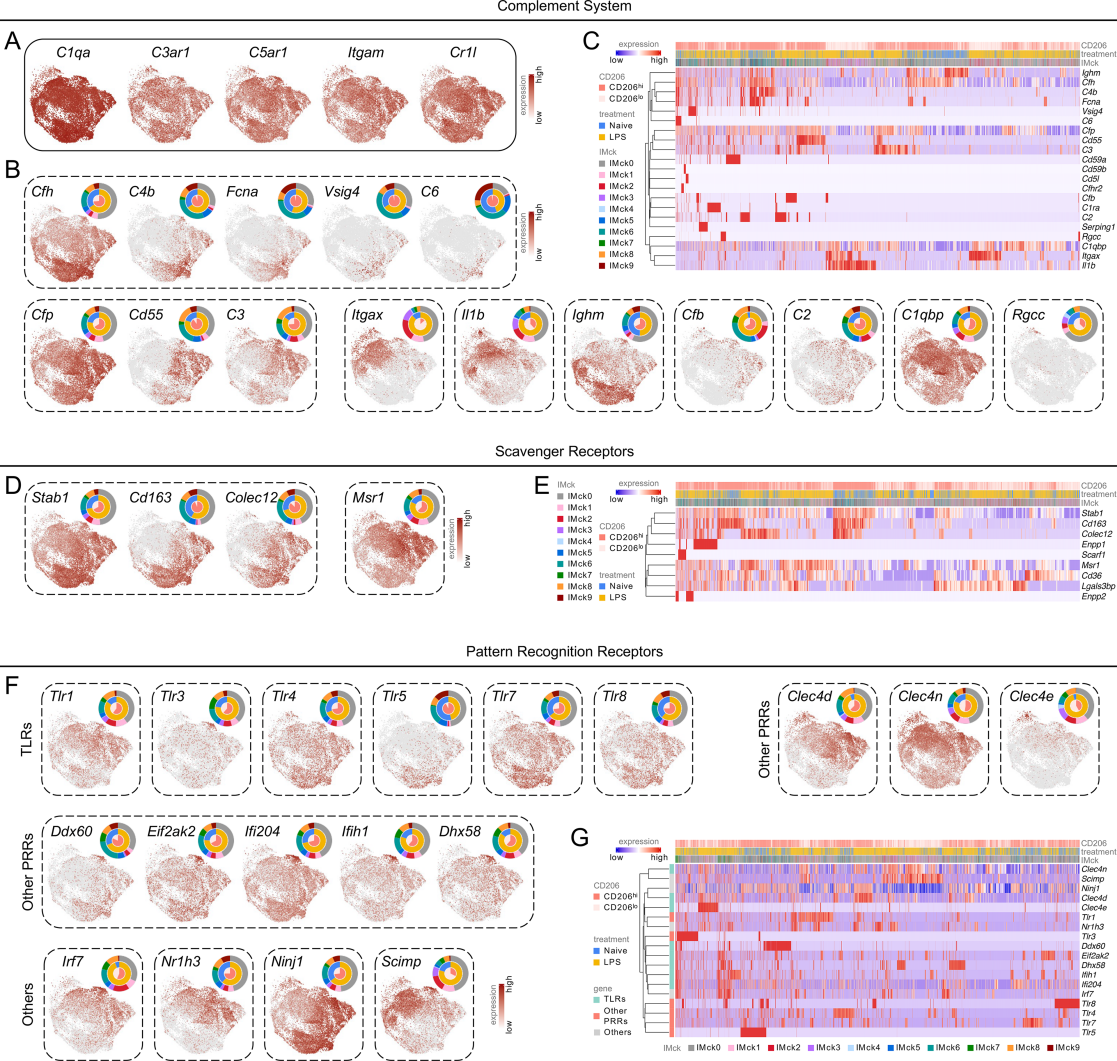
Innate immune function gene expression among IMs. **(A)** Feature plots displaying expression of complement system genes universally expressed by IMs. **(B)** Feature plots displaying the differential expression of complement system genes, with categories annotated as a multilayer pie chart. **(C)** Heat map visualizing the expression of complement system genes in individual IMs, with categories annotated on top. **(D)** Feature plots displaying the differential expression of scavenger receptor genes, with categories annotated as a multilayer pie chart. **(E)** Heat map visualizing the expression of scavenger receptor genes in individual IMs, with categories annotated on top. **(F)** Feature plots displaying the differential expression of pattern recognition receptor genes, with categories annotated as a multilayer pie chart. Genes are shown by different classes. **(G)** Heat map visualizing the expression of pattern recognition receptor genes in individual IMs, with categories annotated on top. Genes are annotated in different classes on the left.

The complement system is central to innate immunity, working with macrophages to remove pathogens, clear damaged cells, and sustain immune balance (48). Indeed, many complement-related genes are robustly, and for some, specifically expressed by IMs (**Figures 3A–C**; **Supplementary Figure S3A–C**). Two of the best-characterized complement receptors on macrophages, CR3 (CD11b/CD18 [*Itgam*/*Itgb2*]) and CR4 (CD11c/CD18 [*Itgax*/*Itgb2*]), bind iC3b to facilitate the clearance of opsonized targets (48). Macrophages also carry C5aR1 (*C5ar1*), the receptor for the potent anaphylatoxin C5a, which serves as a marker to distinguish monocytes and macrophages from dendritic cells (**Figure 3A**) (3, 49). Previous findings indicated that IMs have unique complement protein profile, including C1q, which effectively distinguishes IMs from AMs in naive conditions by flow cytometry (3).

A comprehensive analysis of complement genes identified *C1qa*, *C1qb*, *C1qc*, *C3ar1*, *C5ar1*, *Itgam*, and *Cr1l* as widely expressed by IMs (**Figure 3A**, **S3A**, **S3C**). Among these, *Cr1l, C3ar1*, *C5ar1*, and *Itgam* are detected in other myeloid lineages and lymphocytes (**Supplementary Figure 3A**). In contrast, *C1qa*, *C1qb*, and *C1qc*—encoding the C1q subunits—remain largely specific to IMs in the naive lung, showing no expression in AMs or other immune cell types, making them robust IM markers at the mRNA level (**Supplementary Figure 3A**).

Several complement genes exhibit differential expression across IM subsets (**Figures 3B, C**; **Supplementary Figure 3B**). Overall, CD206^hi^ IMs broadly express complement-related genes such as *Cfp*, *Cd55*, and *C3* (**Figure 3B**). Specifically naive CD206^hi^ IMs (or IMck6 and IMck9) upregulate *Cfh*, *C4b*, *Fcna*, *Vsig4*, and *C6*, whereas CD206^lo^ IMs (or IMck1, IMck2, and IMck3) express *Itgax* and *Il1b*. Unexpectedly, *Ighm* (a gene classically associated with B cells and a key initiator of the classical complement cascade), is expressed in naive mouse IMs. However, IgM protein is not detected in these cells, likely due to the absence of light chains (data not shown). Additionally, genes such as *Cfb*, *C2*, and *C1qbp* increase with LPS stimulation, while *Rgcc* is selectively expressed by IMck4, which links sublytic membrane attack complex (MAC) formation to cell proliferation and inflammation (50). Taken together, both universal and subset-specific complement gene expression suggest that IMs not only harness complement pathways to enhance pathogen clearance but also exhibit distinct complement-driven regulatory programs across subsets.

Together, these findings suggest that IM subsets differ in complement-associated programs that may contribute to pathogen sensing, debris clearance, and immune regulation.

### Scavenger receptor and PRR gene expression across IM subsets

Macrophages rely on a wide range of receptors to detect and respond to potential threats (51). Two major receptor classes essential for macrophage functionality include scavenger receptors and pattern recognition receptors (PRRs). CD206^hi^ IMs exhibit high expression of multiple scavenger receptors targeting oxidized LDL (oxLDL) and apoptotic cells (**Figures 3D, E**; **Supplementary Figure S3D**). These include Stabilin-1 (*Stab1*), which facilitates the removal of oxLDL, extracellular matrix components, or advanced glycation end products (AGE) (52, 53); CD163 (*Cd163*), which binds haptoglobin–hemoglobin complexes to mitigate oxidative stress (54, 55); and COLEC12 (*Colec12*), which detects oxidized phospholipids on apoptotic cells, promoting efficient debris clearance (56). Beyond subset-specific differences, activation status also modulates scavenger receptor gene expression. For instance, *Msr1*, which encodes the macrophage scavenger receptor 1—a key receptor for oxLDL and AGE (57)—is predominantly expressed in LPS-stimulated IMs. (**Figures 3D, E**). The differential expression of these receptors suggests that IM subsets may be equipped for distinct roles in maintaining homeostasis, mounting immune responses, and regulating metabolic processes. Notably, many scavenger receptors (including *Colec12* and *Msr1*) also recognize bacterial ligands (58), potentially connecting them to another major function of macrophages: pathogen recognition and clearance.

PRRs are innate immune sensors that detect pathogen-associated molecular patterns (PAMPs) or danger-associated molecular patterns (DAMPs). By expressing various classes of PRRs and PRRs-related genes, macrophages serve as the first line of defense against a range of infections and tissue damage (**Figures 3F, G**, **S4**). Among those, Toll-like receptors (TLRs), a major PRR family, exhibit distinct expression patterns within IM populations (**Figure 3F**). CD206^hi^ IMs prominently express *Tlr4*, *Tlr5*, *Tlr7*, and *Tlr8*, which decrease upon TLR4-ligand stimulation (**Supplementary Figure 4A**). Additionally, bacterial LPS exposure induces *Tlr1* expression, potentially priming IMs for further bacterial encounters. Beyond TLRs, bacterial-sensing C-type lectin genes (*Clec4d*, *Clec4n*, *Clec4e*) are upregulated in LPS-stimulated IMs, with *Clec4e* showing the highest expression in IMck4.

The viral double-stranded RNA sensor *Tlr3* is restricted to IMck7, aligning with its increased *Irf7* and other related PRR genes such as *Ddx60* (a RIG-I–like receptor cofactor (59)), *Eif2ak2* (a dsRNA sensor (60)), *Ifi204* (a DNA sensor (61)), *Ifih1* (MDA5), *Dhx58* (a regulator of RIG-I/MDA5 (62)), and others (**Figures 3F, G**; **Supplementary Figures 4E, F**). Stimulation of IMs induces the expression of *Nr1h3*, encoding the nuclear receptor transcription factor LXRα, which regulates anti-inflammatory responses (63). Activation of TLR signaling pathways also primes inflammasomes, subsequently triggering pyroptosis—a highly inflammatory form of programmed cell death (64). During pyroptosis, membrane rupture is mediated by Ninjurin-1 (65), whose gene, *Ninj1*, is prominently expressed in CD206^hi^ IMs, further suggesting a reinforcement of the inflammatory capacity of these subsets alongside their robust expression of multiple TLR receptors. Conversely, the TLR adaptor gene *Scimp* exhibits the highest expression in CD206^lo^ IMs, highlighting an opposite expression pattern.

These data suggest that IM subsets possess distinct innate-recognition programs, with CD206hi and CD206lo IMs differing in their expression of scavenger receptors, TLRs, C-type lectins, and cytoplasmic PRR-associated genes.

### Spatial localization of CD206^hi^ and CD206^lo^ IMs

**Figure 4:**
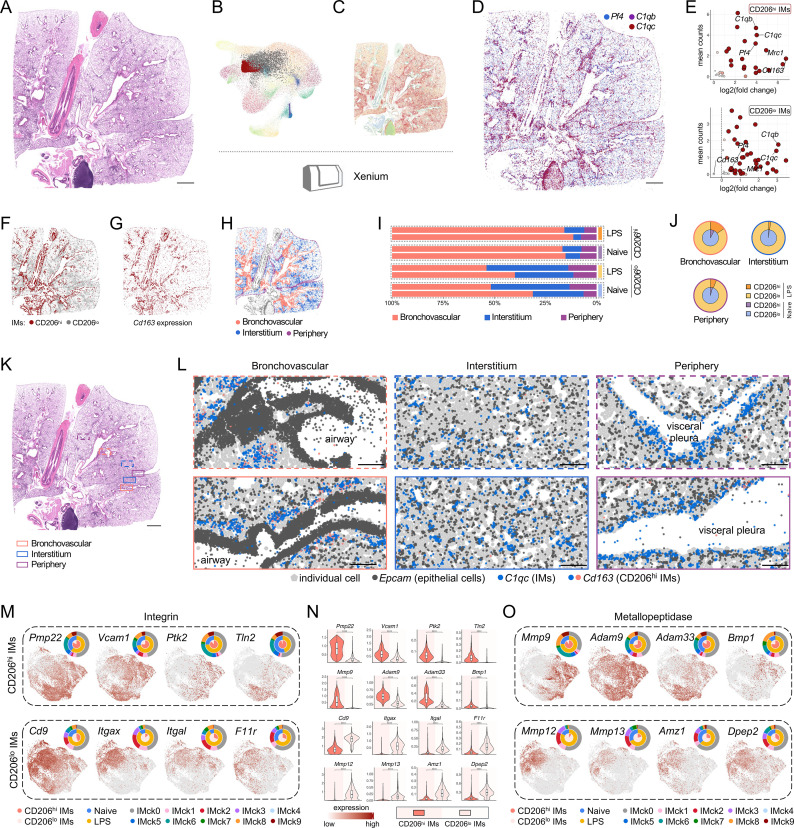
Spatial localization of CD206^hi^ and CD206^lo^ IMs. A–C. Representative H&E-stained image **(A)**, Xenium UMAP plot **(B)**, and Xenium image **(C)** illustrating graph-based clustering of lung cells 24 hours post-LPS treatment (scale bars, 1,000 µm). **(D)** Representative Xenium image showing the expression of IM marker genes in the sample, including *Pf4*, *C1qb*, and *C1qc* (scale bars, 1,000 µm). **(E)** Scatter plot depicting marker gene expression for graph-based CD206^hi^ and CD206^lo^ IM clusters in LPS-treated Xenium samples, specifically *Pf4*, *C1qb*, *C1qc*, *Mrc1*, and *Cd163*. **(F)** Representative Xenium image displaying spatial localization of CD206^hi^ and CD206^lo^ IM clusters identified through graph-based clustering. **(G)** Representative Xenium image illustrating *Cd163* expression specifically within IMs. **(H)** Representative Xenium image highlighting distinct IM localizations in the lung, including bronchovascular bundles, interstitial areas, and peripheral regions. **(I)** Percentage bar graph showing the distribution of IM subsets across different localizations for each sample. **(J)** Multilayer pie chart representing IM composition in various localizations, with the inner layer corresponding to naive samples and the outer layer corresponding to LPS-treated samples. **(K)** H&E-stained image highlighting representative areas for different localizations (scale bars, 1,000 µm). **(L)** Zoomed-in images from representative areas shown in **(K)**, illustrating differential localization patterns of CD206^hi^ and CD206^lo^ IM subsets (scale bars, 1,000 µm). **(M)** Feature plots displaying the differential expression of integrin genes within CD206^hi^/CD206^lo^ IMs, with categories annotated as a multilayer pie chart. Genes are shown by different classes. **(N)** Violin plot comparing integrin and metallopeptidase gene expression across CD206^hi^/CD206^lo^ IMs. Within each violin, a box plot spans the interquartile range (25th to 75th percentiles) with a horizontal line at the median; whiskers extend to 1.5 × the interquartile range. P values calculated using Wilcox test. *P < 0.05; **P < 0.01; ***P < 0.001; ****P < 0.0001; nonsignificant results not shown. **(O)** Feature plots displaying the differential expression of metallopeptidase genes within CD206^hi^/CD206^lo^ IMs, with categories annotated as a multilayer pie chart. Genes are shown by different classes.

**Figure 5:**
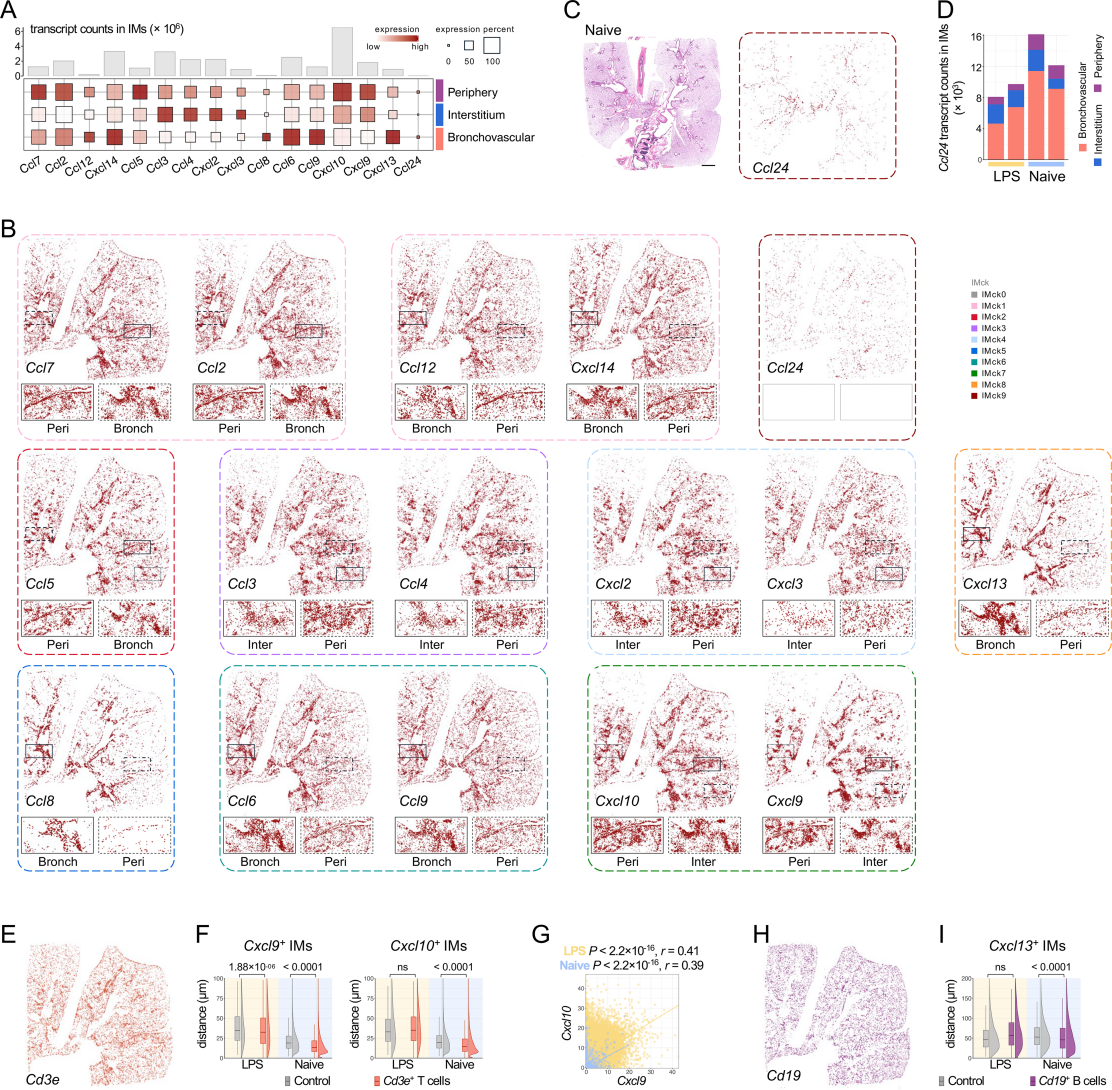
Spatial localization of chemokine-expressing IMck subsets. **(A)** Dot plot highlighting differential localization patterns and total transcript counts of chemokine expression within LPS-treated lung IMs. **(B)** Representative Xenium image showing chemokine gene expression in LPS-treated lung IMs. Black boxes indicate representative regions for different localizations: solid boxes highlight the localization category with the highest chemokine expression, dotted boxes indicate the localization category with the second-highest expression, and lighter dotted boxes indicate the localization category with the lowest but still enriched expression. Zoomed-in images of these representative areas are shown at the bottom. Chemokine expression combinations defining IMck subsets are indicated by colored dotted boxes corresponding to each IMck subset. *Ccl24* exhibits minimal expression across all three lung localizations. **(C)** Representative H&E-stained image (left panel) and Xenium image of lung IM *Ccl24* expression (right panel) from naive lung tissue (scale bars, 1,000 µm). **(D)** Stacked bar graph displaying the distribution of total IM *Ccl24* transcript counts across different localizations for each sample. **(E)** Representative Xenium image showing *Cd3e* expression in the LPS-treated lung. **(F)** Distance analysis comparing spatial distances of *Cxcl9*+ or *Cxcl10*+ IMs to *Cd3e*+ T cells versus control pseudo-T cells, assessing potential enrichment of T cells close to chemokine-expressing IMs. *P values* calculated using Wilcox test. Ns., not significant. **(G)** Scatter plot illustrating the Xenium co-expression patterns of *Cxcl9* and *Cxcl10* transcripts within IMs from naive and LPS-treated lungs. Statistical analysis was performed using Pearson’s correlation coefficient (*r*) and associated *P values*. **(H)** Representative Xenium image showing *Cd19* expression in the LPS-treated lung. **(I)** Distance analysis comparing spatial distances of *Cxcl13*+ IMs to *Cd19*+ B cells versus control pseudo-B cells, assessing potential enrichment of B cells close to *Cxcl13*+ IMs. *P values* calculated using Wilcox test. Ns., not significant.

As tissue-resident immune cells, the transcriptomic profiles of IMs are closely linked to their functional heterogeneity. Indeed, earlier research suggests distinct niches for IM subsets (8, 22–24, 66). To investigate this further, we used the mRNA-probe-based 10x Xenium spatial transcriptomics approach, allowing precise integration with existing scRNA-seq data from the same experimental conditions (LPS-treated IMs: **Figures 4**, **5**, naive IMs: **Supplementary Figure 7**, all IMs: **Supplementary Figure 5**). Graph-based clustering was applied to the spatial transcriptomics data to identify cell types (**Figures 4A–C**, **S5**). IMs were defined based on robust markers included in our panel, such as C1q-related genes, *Pf4*, *Folr2*, *Mrc1*, and *Cd163* among others (3) (**Figure 4D**, **S5**). Consistent with scRNA-seq findings, both CD206^hi^ and CD206^lo^ IMs showed strong and specific expression of *C1qb*, *C1qc*, and *Pf4*, with differing levels of *Mrc1* enrichment (**Figure 4E**). Notably, CD206^hi^ IMs uniquely expressed high levels of *Cd163*, while CD206^lo^ IMs exhibited negative enrichment, suggesting that *Cd163* may serve as a more robust surrogate marker than *Mrc1* for distinguishing these subsets (**Figures 4E–G**). In the Xenium dataset analyzed here, spatial analysis revealed that IMs primarily localize to three distinct lung regions: (1) bronchovascular bundles—containing bronchial epithelium, vasculature, and nerve bundles; (2) interstitium; and (3) the peripheral region adjacent to the visceral pleura (**Figure 4D, F-H**) (6). Within these anatomical niches, CD206^hi^ and CD206^lo^ IMs exhibited preferential distribution patterns in our samples: CD206^hi^ IMs were enriched around bronchovascular bundles, whereas CD206^lo^ IMs were detected across all three lung compartments (**Figures 4H–L**). Interestingly, in our Xenium samples, CD206^lo^ IMs consistently outnumbered CD206^hi^ IMs across all niches, including the CD206^hi^ IM-rich bronchovascular bundle region, where CD206^hi^ IMs comprised only approximately 16% of the total IM population following LPS stimulation.

This distinct localization prompted further examination of genes associated with spatial positioning. Indeed, multiple integrin and metallopeptidase genes exhibit distinct expression patterns between CD206^hi^ IMs and CD206^lo^ IMs (**Figures 4M–O**, **S6**). CD206^hi^ IMs strongly express *Pmp22*, *Vcam1*, *Ptk2*, and *Tln2*, which encode key mediators of integrin signaling and adhesion (**Figures 4M, N**, **S6B**). VCAM1, for instance, is a vascular cell adhesion molecule that facilitates leukocyte-endothelial interactions (67). CD206^hi^ IMs also express *Lamb2*, a basement membrane component (68) (**Supplementary Figures 6A, B**). These factors may partly explain their clustering near bronchovascular regions. Additionally, CD206^hi^ IMs exhibit high levels of metallopeptidases such as *Mmp9*, *Adam9*, *Adam33*, and *Bmp1*, which remodel extracellular matrix (ECM) proteins like collagen to enable tissue repair, immune cell migration, and inflammation (**Figures 4N, O**, **S6B**). Such abilities to remodel ECM around blood vessels are consistent with possible chemoattractant roles under both steady-state and stimulated conditions, contributing to the formation of inducible bronchus-associated lymphoid tissue (iBALT) (3), a process involving significant tissue restructuring.

Meanwhile, CD206^lo^ IMs express a different set of integrin and metallopeptidase genes, including *Cd9*, *Itgax*, *Itgal*, *F11r*, *Mmp12*, *Mmp13*, *Amz1*, and *Dpep2*, reflecting their potential specialized roles in adhesion and ECM remodeling (**Figures 4M, N**, **S6A–B**). CD11a (*Itgal*) pairs with CD18 to form LFA-1, crucial for leukocyte adhesion to endothelium and immune synapse formation (69). *F11r* encodes JAM-A, supporting both tight junction integrity and leukocyte transmigration (70). Additionally, *Emilin1*, specifically expressed by CD206^lo^ IMs, contributes to elastic fiber organization, influencing tissue stiffness (71)—an essential aspect of lung function (**Supplementary Figures 6A, B**). However, integrin and metallopeptidase gene expression also varies with activation states (e.g., *Itgb5* in the naive state versus *Itga5* under LPS stimulation, **Supplementary Figures 6C, D**). Further investigation is needed to delineate the precise molecular cues directing spatial localization and to understand the functional significance behind each subset’s placement.

Together, these findings from our Xenium samples suggest that CD206^hi^ and CD206^lo^ IMs occupy partially distinct anatomical niches and express candidate adhesion and remodeling genes that may be associated with their spatial organization.

### Spatial localization of chemokine-expressing IMck subsets

With chemoattraction being one of the major functions of IMs, we examined chemokine expression at the spatial level (LPS-treated IMs: **Figure 5**, naive IMs: **Supplementary Figure 7**). The division of labor for chemokine expression extends to the anatomical niche localization of LPS-stimulated IMs, closely mirroring the chemokine expression patterns observed in scRNA-seq-defined IMck subsets (**Figures 5A, B**). IMck1 chemokines and *Ccl5* exhibit enrichment at the bronchovascular bundles (predominantly *Ccl12* and *Cxcl14*) and the periphery (primarily *Ccl7*, *Ccl2*, and *Ccl5*). *Ccl3*, *Ccl4*, *Cxcl2*, and *Cxcl3* (IMck3 and IMck4 chemokines) display the most concentrated expression in the interstitium, forming distinct aggregated expression clusters, as exemplified by the zoomed-in interstitial image of their expression (**Figure 5B**). *Ccl8* (IMck5) and *Cxcl13* (IMck8) display similar patterns, with enrichment in bronchovascular bundles and minimal expression in other niches. IMck6 chemokines *Ccl6* and *Ccl9* largely follow the pattern of *Ccl8* and *Cxcl13* but exhibit slightly higher expression in other niches. IMck7 chemokines *Cxcl10* and *Cxcl9* have distinct expression patterns, with the highest expression in peripheral IMs, while also forming localized expression clusters in the interstitium, similar to *Ccl3* and *Ccl4*. This pattern is likely driven by local proinflammatory feedback loops. *Cxcl10* and *Cxcl9* are examples of chemokine genes that show strong expression across all localizations, as indicated by their enrichment not only in the interstitium and periphery but also at the branch points of the bronchial airway. Notably, their branch point pattern is distinct from other bronchovascular bundle-enriched chemokines, such as *Cxcl13*, which exhibits high expression along the entire bronchial tree.

Consistent with our observations of *Ccl24* expression in the scRNA-seq dataset, *Ccl24* exhibited low expression with no discernible spatial pattern in the LPS-treated Xenium sample (**Figures 5A, B**). In contrast, naive lungs expressed higher levels of *Ccl24*, primarily along the bronchial airways (**Figures 5C, D**). The spatial expression of other chemokines in the naïve lung remained largely consistent with their LPS-stimulated patterns, with the exception of a significant reduction in interstitial and peripheral expression. This reduction led to limited detection of proinflammatory chemokines (*Ccl3*, *Ccl4*, *Cxcl2*, *Cxcl3*, *Cxcl10*, and *Cxcl9*) and an accentuated bronchovascular localization of other chemokines (**Supplementary Figures 7A, B**).

One of the key advantages of spatial transcriptomics is its ability to resolve cell-cell proximity. Leveraging this, we further investigated the spatial relationship between chemokine-expressing IMs and their corresponding recruited leukocytes (**Figures 5E–I**, **Supplementary Figures 7C, D**). Compared to control (randomized pseudo-T cells), we found that *Cd3e*^+^ T cells were preferentially enriched near *Cxcl9*^+^/*Cxcl10*^+^ IMs (**Figures 5E, F**). The spatial expression pattern of *Cd3e* further suggests that chemokine-expressing IMs and T cells are spatially associated near the bronchovascular bundles and periphery. Notably, *Cxcl9* and *Cxcl10* were strongly co-expressed in both naïve and LPS-treated IMs, supporting the previously proposed coordinated expression (**Figure 5G**). Similarly, we observed *Cd19*^+^ B cells in closer proximity to *Cxcl13*^+^ IMs in the naïve lung, further supporting a spatial association between chemokine-expressing IMs and lymphocyte positioning. (**Figures 5H, I**).

Together, these spatial analyses suggest that, in the Xenium samples analyzed here, chemokine-expressing IMs are organized across distinct lung niches and are associated with the local positioning of selected lymphocyte populations.

**Figure 6:**
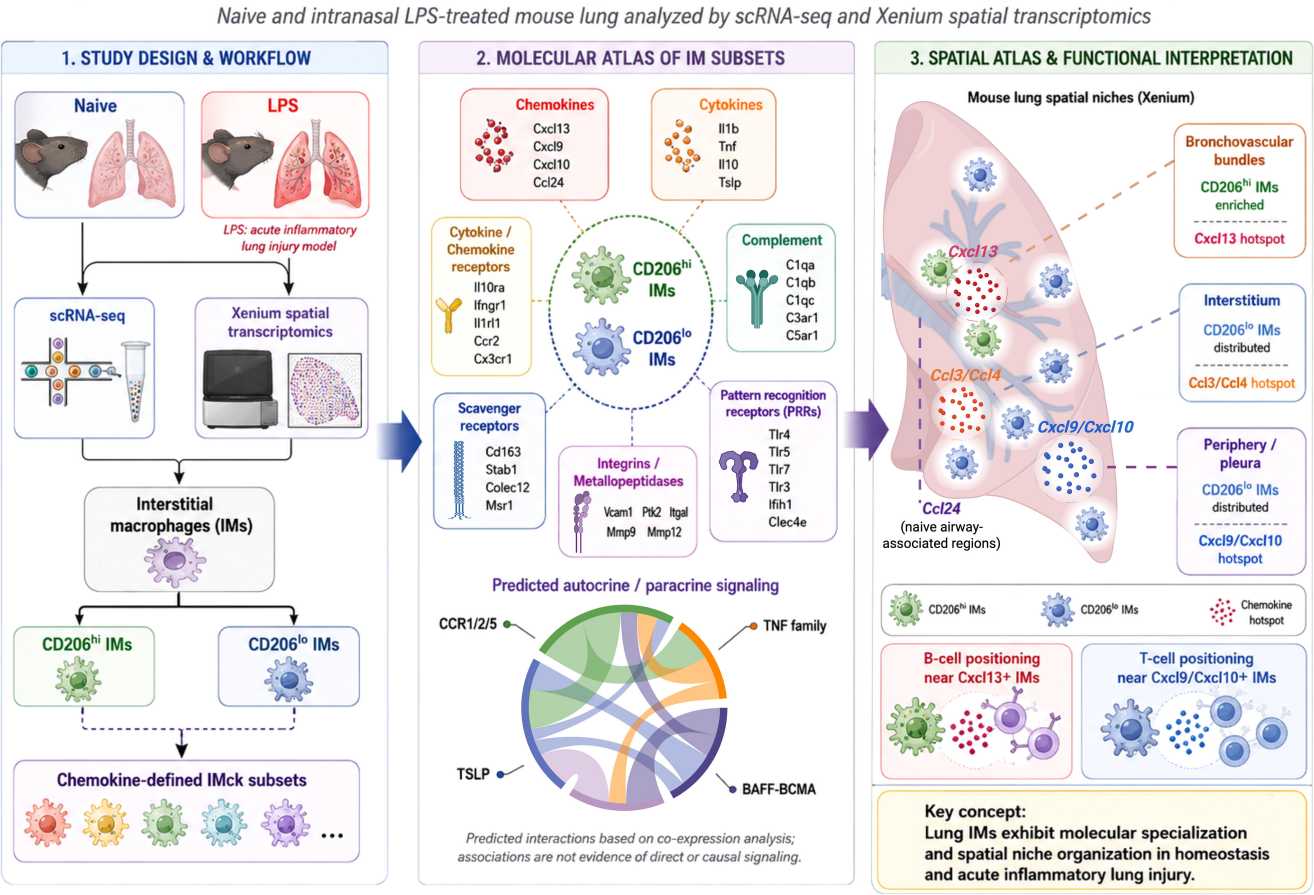
Summary schematic of the molecular and spatial atlas of lung interstitial macrophage subsets. Naive and intranasal LPS-treated mouse lungs were analyzed by scRNA-seq and Xenium spatial transcriptomics to characterize tissue-resident IMs. The schematic summarizes the major analytical components of the study, including CD206^hi^ and CD206^lo^ IMs, chemokine-defined IMck subsets, cytokines, cytokine/chemokine receptors, complement genes, scavenger receptors, PRRs, integrins, metallopeptidases, predicted autocrine/paracrine ligand-receptor interactions, and spatial localization across bronchovascular bundles, interstitium, and peripheral/pleural regions. The predicted interaction network summarizes candidate ligand-receptor relationships inferred from gene-expression patterns and should not be interpreted as direct evidence of active signaling. Spatial features summarize the main Xenium-based observations, including niche-associated enrichment of CD206^hi^ and CD206^lo^ IMs, spatially patterned chemokine expression, and associations between chemokine-expressing IMs and local lymphocyte positioning. Overall, the schematic highlights the major concept that lung IM subsets exhibit molecular specialization and spatial niche organization in homeostasis and acute inflammatory lung injury.

To integrate these findings, we generated a summary schematic highlighting the study design, IM subset framework, major molecular systems analyzed, predicted cytokine/chemokine interaction networks, and Xenium-defined spatial organization of lung IM subsets (**Figure 6**).

## Discussion

Our study expands the current understanding of lung IM heterogeneity by detailing their transcriptional diversity, spatial organization and proposed functional specialization. While AMs remain a central focus in pulmonary immunology, IMs are gaining increasing attention due to their critical roles in immune surveillance, tissue repair, and inflammation modulation (1–3). By integrating scRNA-seq and Xenium spatial transcriptomics, we showed that both primary IM subsets—CD206^hi^ and CD206^lo^—and the IMck subsets (IMck0–9) exhibit heterogeneous gene expression patterns across cytokine systems, complement pathways, scavenger receptors, PRRs, and spatial localization within the lung (**Figure 6**). Importantly, our analysis includes both naive lungs and lungs exposed to LPS, allowing us to compare steady-state IM organization with IM responses during acute inflammatory stimulation. Pulmonary LPS administration is widely used as an experimental model with key inflammatory features relevant to ARDS, including acute pulmonary inflammation, inflammatory-cell recruitment, epithelial/endothelial barrier disruption, and neutrophilic injury (26–28). Although this model does not reproduce the full complexity of human ARDS, it provides a useful framework for identifying IM molecular and spatial programs that may become relevant during acute inflammatory lung injury.

Consistent with previous studies (22–24), we found that CD206^hi^ IMs preferentially express genes associated with phagocytosis and tissue repair, including scavenger receptors and type 2 cytokines, whereas CD206^lo^ IMs exhibit a transcriptional profile consistent with antigen presentation and pro-inflammatory signaling, characterized by high expression of genes like *Il1b* and *Tnf*. Alternatively, IMs are marked by distinct chemokine expression patterns, where each IMck subset possessed a unique “core” chemokine signature, suggesting a division of labor in immune cell recruitment. For instance, the *Cxcl13*-expressing IMck8 subset, primarily enriched among CD206^hi^ IMs, supports the organization of inducible bronchus-associated lymphoid tissues (iBALTs) (3). Such compartmentalized chemokine production suggests that distinct IM subsets are equipped to orchestrate local immune cell recruitment and positioning in response to specific environmental cues (e.g., infection or tissue injury).

The diverse array of cytokine and receptor genes expressed by IMs, including IL-10, TSLP, IFN-γ receptors, and members of TNF superfamily, suggests that these cells may be capable of complex immune interactions. Notably, the strong expression of receptors for both pro-inflammatory signals (e.g., *Ifngr1*) and anti-inflammatory signals (e.g., *Il10ra*, *Il1rl1* [ST2]) in naive IMs underlines their potential to fine-tune local immune responses. This is particularly relevant in the lung, where macrophages must rapidly transition between homeostatic and inflammatory states. Moreover, the upregulation of genes such as *Il1rn* (IL-1 receptor antagonist) in stimulated IMs suggests that they possess intrinsic mechanisms to restrain excessive inflammation. Together, these findings expand our understanding of IM versatility, suggesting that these cells can dynamically shift across a spectrum of activation states *in situ*.

Additionally, we provide a comprehensive map of predicted cytokine/chemokine autocrine signaling within IMs. A complex chemotaxis network involving *Ccr1*, *Ccr2*, and *Ccr5* emerged as the most enriched autocrine signaling system, suggesting a candidate role in regulating IM localization and migration that requires future functional testing. The TNF family autocrine system was also highly enriched, though certain components, such as *Tnfrsf17*, remain relatively unexplored in macrophages. Beyond the autocrine effects of cytokines and chemokines, IM subsets also interact through various molecular pathways, including the APOE-TREM2 (*Apoe*-*Trem2*) axis (**Supplementary Figures 8A–D**), which has been implicated as a major regulator of microglial function in neurodegenerative diseases such as Alzheimer’s (64). Additionally, enriched APP-CD74 and PPIA-BSG interactions may contribute to IM activation and pro-inflammatory responses (72, 73) (**Supplementary Figures 8A, B**).

We also noted a pronounced expression of complement-related genes (*C1qa*, *C1qb*, *C1qc*, *C3ar1*, *C5ar1*) in IMs. While alveolar macrophages are traditionally associated with surfactant clearance and homeostasis, our data indicate that IMs may be the principal complement-expressing macrophage population in the steady-state lung (3). This robust complement signature suggests that IMs may be equipped to opsonize and clear pathogens or apoptotic cells, while also coordinating adaptive immunity via complement-mediated signals. Such functionality, combined with distinct expression of scavenger receptors and pattern recognition receptors (PRRs), supports IMs’ essential role as innate sentinels and mediators of lung integrity.

Using 10x Xenium spatial transcriptomics, we investigated the spatial properties of naive and LPS-treated lung IMs. In our Xenium dataset, IMs were primarily detected across three distinct anatomical niches in the lung: the bronchovascular bundles, interstitium, and periphery. Further analysis revealed that CD206^hi^ and CD206^lo^ IMs show different niche-enrichment patterns in the analyzed samples. CD206^hi^ IMs cluster near bronchovascular bundles, expressing key adhesion molecules (e.g., *Vcam1*, *Ptk2*) and metallopeptidases (e.g., *Mmp9*, *Bmp1*) that may facilitate tissue remodeling and leukocyte recruitment at sites where blood vessels and airways converge. In contrast, CD206^lo^ IMs were more broadly distributed, including regions near the visceral pleura, and exhibited a distinct integrin/metallopeptidase signature (e.g., *Itgal*, *Mmp12*). Previous studies have relied primarily on immunofluorescent staining to study IM localization, using markers such as TREM2, CD169/CD11c and LYVE1/CX3CR1 (8, 22, 23, 66). However, this approach is constrained by its reliance on a limited set of markers, variability in marker robustness, antibody performance, and background noise. By contrast, Xenium detects mRNA expression, allowing closer alignment with scRNA-seq data and providing a more accurate representation of IM heterogeneity. However, achieving true single-cell resolution with Xenium remains challenging due to difficulties in precisely determining cell boundaries across all three dimensions. And whether these niche-specific distributions arise from distinct ontogeny, differential exposure to tissue-derived signals, or a combination of both remains an area for future exploration. Another interesting methodological observation is the overrepresentation of CD206^lo^ IMs (excluding recMacs) compared to CD206^hi^ IMs in both LPS-treated and naïve lungs. This contradicts the flow cytometry data, where we typically observe slightly more CD206^lo^ compared to CD206^hi^ IMs. Given their distinct localization patterns, we hypothesize that the apparent underrepresentation of CD206^lo^ IMs in flow cytometry is due to insufficient digestion or digestion-induced damage of CD206^lo^ IMs in the lung. This finding highlights the importance of studying immune cells *in situ* with minimal perturbation.

Beyond the analysis of CD206^hi^/CD206^lo^ IMs, we also utilized spatial transcriptomics to examine chemokine expression in lung IMs. Consistent with scRNA-seq data, our findings suggest that different chemokines exhibit coordinated expression patterns and preferential localization in LPS-treated lungs. For example, *Ccl8* and *Cxcl13* are enriched in the bronchovascular region. Typical proinflammatory chemokines may contribute to a local proinflammatory feedback loop, amplifying inflammation and leading to intense localized expression clusters, such as the interstitial expression of *Ccl3*, *Ccl4*, *Cxcl10*, and *Cxcl9*.

Interestingly, proximity analysis revealed that in the steady state, chemokine-expressing IMs and their corresponding leukocytes tend to co-localize. However, under LPS stimulation, this enrichment becomes less pronounced. We hypothesize that this may be time-dependent, as our samples were collected 24 hours after *i.n.* LPS stimulation, and the observed sparse localization may result from acute endothelial leakage. At later time points, as vascular integrity stabilizes following the acute phase of inflammation, we expect recruited leukocytes to more effectively co-localize with their corresponding IMs.

The classification of IMck is based on chemokine expression (3), yet maintains strong compatibility with other gene families mentioned above. Unlike the rest of IMck clusters, IMck0 comprises multiple unbiased clusters identified through high-resolution analysis (**Supplementary Figure 8E**). Differentially expressed gene analysis revealed some heterogeneity within IMck0, such as the *Hmox1*-expressing cluster 6; however, most clusters lack distinct DEGs that distinguish them from others (**Supplementary Figures 8E–G**, **Supplementary Table 3**), supporting the subsetting approach used in this study.

Our previous study analyzed growth factors and their receptors in IM populations and found no enriched expression, except for *Tgfb1* and *Igf1* (3). We also observed *Nes*, a neurotrophic factor, was expressed by CD206^lo^ IMs (3), a subset known to wrap around neurons (71), suggesting a functional interaction or potential *Nes* mRNA uptake from neighboring neurons.

Although our analysis reveals a great degree of heterogeneity among IM subsets, several limitations should be considered. First, conclusions in this study are based on transcriptional profiles, predicted ligand-receptor interactions, or spatial proximity, and therefore should be interpreted as hypotheses rather than direct functional proof. Similarly, CellPhoneDB analysis identifies candidate ligand-receptor interactions but does not demonstrate active signaling. The Xenium analysis was performed with two mice per group and should therefore be interpreted as an exploratory spatial atlas rather than definitive evidence of invariant localization patterns. Although the observed spatial patterns were consistent with the transcriptional profiles identified by scRNA-seq, additional biological replicates and disease models will be needed to determine how generalizable these niche-localization patterns are across inflammatory contexts. In addition, all experiments were performed in female mice; therefore, potential sex-dependent differences in IM biology were not addressed. Functional validation also remains limited due to the lack of subset-specific genetic mouse models for selective manipulation. Furthermore, whether the distinct molecular profiles of IMs are developmentally preprogrammed or shaped by local signals, such as cytokines from stromal or epithelial cells, remains incompletely understood. Single-cell multi-omic approaches, advanced lineage tracing, and *in vivo* perturbation experiments will be essential for dissecting how niche signals shape IM subsets during homeostasis and disease. Lastly, similar to AMs (9), understanding the parallels and differences between human and mouse IMs will be key to determining the translational relevance of our findings.

In summary, our findings map the complex molecular and spatial heterogeneity of lung IMs and identify candidate programs through which specialized subsets may contribute to tissue homeostasis, immune regulation, and host defense through the coordinated action of cytokines, chemokines, complement factors, scavenger receptors, PRRs, integrins, and MMPs (**Figure 6**). This work establishes the foundation for future investigations aimed at targeting IM functions in lung pathogenesis.

## Materials and methods

### Mice

C57BL/6NCrl (C57BL/6) wild-type mice were purchased from Charles River Laboratories/NCI. All mice were bred in-house. Mice were genotyped or phenotyped before studies and used at 6–12 weeks of age; all experiments were performed on age-matched cohorts. Female mice were used in each condition of each experiment. Mice were kept in specific-pathogen-free conditions maintained by the Dartmouth Hitchcock Medical College. Facilities were maintained at an ambient temperature of 23–24 °C with a 12-h light–dark cycle. Mice had access to food and water ad libitum.

### *In vivo* IM stimulation

For stimulation, mice were anesthetized with Avertin (Sigma-Aldrich) and administered 25 μg LPS (Sigma-Aldrich) in 50 μl PBS intranasally (*i.n.*). Control mice received 50 μl PBS without LPS. Mice were euthanized 24 hours post-instillation for analysis of *in vivo*-stimulated IMs in all experiments.

### Flow cytometry

The mice were euthanized and the lungs were perfused, finely minced, and digested with 2.5 mg/ml collagenase D (Roche) and 400 μg/mL of Liberase ™ solution (Roche) for 30 min at 37 °C. Single-cell suspensions were generated by repeated glass pipetting and filtration. Single-cell suspensions were resuspended in FACS buffer containing HBSS (Corning) with 0.3 mM EDTA (Gibco) and 0.2% FBS (Corning) and stained for 30 min with antibody master mix. The viability dye DAPI (Sigma) was added before sample acquisition on a BD Symphony A3 analyzer (BD Biosciences). Data were analyzed using FlowJo (Tree Star).

### Xenium data and analysis

#### Mouse treatment

Two groups of C57BL/6 mice (n = 2 per group) were used for cell collection: Group 1 (Naive): No treatment, expected to contain only steady-state IMs; Group 2 (LPS): LPS (Sigma-Aldrich) administered intranasally (*i.n.*) at 10 μg in 50 μl sterile PBS (Corning) 24 h before collection, expected to contain stimulated IMs and recruited monocytes.

#### Sample processing

For Xenium spatial transcriptomics (10x Genomics), mice were perfused with 10% Neutral Buffered Formalin (NBF) to remove circulating blood. The lungs were then inflated with NBF to preserve tissue architecture and fixed by submersion in 10% NBF for 12 hours at room temperature. After fixation, lung tissues were processed for paraffin embedding, and formalin-fixed paraffin-embedded (FFPE) blocks were prepared. Sections were then cut at 5um thickness onto Xenium slides in the Pathology Shared Resource at Dartmouth (RRID: SCR_023479) according to 10x Genomics protocol CG000580). Slides were then transferred to the Genomics and Molecular Biology Shared Resource (RRID: SCR_021293) and processed following the manufacturer’s instructions for FFPE tissue sections (Protocol: CG000581) followed by probe hybridization, ligation and amplification (Protocol: CG000582). Slides were run on a Xenium Analyzer instrument running Xenium instrument software version 2.0.1.0 and On-Board Analysis software version 2.0.0.10 to produce the output data bundle used for downstream analysis. Following the Xenium run, slides were H&E stained on a Sakura Tissue-Tek Prism stainer and whole slide imaging conducted at 40x magnification using an Aperio GT450 instrument (Leica). Xenium spatial transcriptomics analysis was then processed and performed using Xenium Explorer 3 (10x Genomics), with cell population identification conducted using R v.4.2 (74).

#### Data preparation

Graph-based clustering results and differentially expressed genes (DEGs) from the 10x Xenium pipeline were utilized for cell-type identification based on known marker combinations, enabling the identification of IM clusters. Spatial localization of IM subsets was explored using the selection tool in Xenium Explorer 3, retrieving relevant cell IDs. Additional analyses were conducted in R v.4.2 (74) using the Seurat Xenium pipeline. To specifically analyze chemokine expression patterns in lung IMs and *Cd3e*/*Cd19* expression in lung T/B cells, connective tissue and heart regions were filtered out based on cell IDs retrieved from Xenium Explorer 3, followed by spatial filtering of molecular coordinates outside targeted polygonal cell boundaries using the custom image.subset function (https://github.com/XinLi-0419/XeniumSpatialMacrophage/).

#### Distance calculation

Distances between chemokine-expressing IMs and T or B cells were computed based on cell centroid coordinates. IMs expressing chemokines (*Cxcl9*, *Cxcl10*, *Cxcl13*) were defined by detection of >0 chemokine gene transcripts, while T/B cells were identified by expression of *Cd3e* or *Cd19* transcripts. The nearest neighbor distances from chemokine-positive IMs to T/B cells were calculated using the get.knnx function from the FNN package (75). As controls, distances were also computed to randomly selected pseudo-T/B cells generated from non-IM cell populations, maintaining equal sample sizes. Statistical significance comparing distances between chemokine-positive IMs to real versus pseudo-T/B cells was assessed using the Wilcoxon rank-sum test (`wilcox.test` in R v.4.2 (74)), testing whether real cell interactions occurred at significantly shorter distances compared to pseudo-cell interactions.

### scRNA-seq data and analysis

The mouse treatment, sample processing, and data preparation for scRNA-seq data used in this study are detailed in (3) and GSE225664.

#### Mouse treatment

Three groups of C57BL/6 mice (n = 2 per group) were used for cell collection: Group 1 (Naive): No treatment, expected to contain only steady-state IMs; Group 2 (LPS): LPS (Sigma-Aldrich) administered intranasally (*i.n.*) at 10 μg in 50 μl sterile PBS (Corning) 24 h before collection, expected to contain stimulated IMs and recruited monocytes; Group 3 (Anti-Gr1 + LPS): Anti-Gr1 (300 μg in 300 μl) administered intraperitoneally, and LPS (10 μg in 50 μl PBS) administered *i.n.*, both 24 h before collection, expected to contain only stimulated tissue-resident IMs (blocked recruitment of blood monocytes).To distinguish intravascular from extravascular leukocytes, mice were injected intravenously with APC-Cy7-conjugated anti-CD45 antibody 5 min before organ collection.

Due to the rarity of IMs in Group 2, data from Group 2 and Group 3 were combined and treated as one LPS group in this study.

#### Sample processing

The mice were euthanized and the lungs were perfused, finely minced, and digested for 30 min at 37 °C. Single-cell suspensions were generated by repeated glass pipetting and filtration. Cells were pelleted by centrifugation at 300g for 5 min at 4 °C, stained with fluorescent-labeled antibodies, and enriched using anti-CD11b MicroBeads (Miltenyi). FACS sorting was performed on a FACS Aria Fusion (BD Biosciences). Sorted cells were centrifuged at 300g for 5 min at 4 °C, and the pellet resuspended in HBSS (Corning) plus 0.5% BSA (Sigma-Aldrich) at ~2.5 × 10^5 cells per ml. Cell quality and viability (>90%) were assessed using a Cellometer K2 (Nexcelom Bioscience). Approximately 6,000 cells were loaded on each channel of the Chromium Next GEM Single Cell 3′ Platform (10x Genomics), with an average recovery of 5,000 cells per channel. Libraries were sequenced on a NextSeq 500/550 (Illumina) at an average depth of 38,000 reads per cell.

#### Data preparation

Data preparation for scRNA-seq is detailed in (3) and GSE225664. Raw sequencing reads were demultiplexed and mapped to the GRCm38 mouse reference genome. Gene expression matrices were generated using CellRanger v.6.1 (10x Genomics). Downstream analyses were performed in R v.4.2 (74) and Python v.3.6. Seurat v.4.3 (76) was used for data integration and analysis, and plots were produced with ggplot2. Following the standard Seurat workflow, the dataset was merged for further analysis.

#### Cell type identification

Cell type identification followed methods detailed in (3, 77–79), wherein IMs were distinguished based on characteristic marker genes and clustering profiles.

#### Differentially expressed genes

DEGs were calculated using Seurat’s FindAllMarkers function in R v.4.2 to analyze differences among IM subsets and treatment groups. The data matrices of the SCT assay were used with a minimum log fold change of 0.25. Only genes detected in more than 25% of cells in either population were considered for the Wilcoxon rank-sum test. DEGs were identified as significantly upregulated if they had a Bonferroni-adjusted P value < 0.05. The resulting gene list was ranked by adjusted P value, and top DEGs were selected for downstream analysis.

#### Gene sets

The gene sets used in this manuscript were obtained from the Gene Ontology (GO) project (80), including GO:0008009 (“chemokine activity”) for chemokine genes; GO:0005125 (“cytokine activity”) for cytokine genes; GO:0004896 (“cytokine receptor activity”) for cytokine and chemokine genes; GO:0006956 (“complement activation”) for complement genes; GO:0005044 (“scavenger receptor activity”) for scavenger receptor genes; GO:0038187 (“pattern recognition receptor activity”), GO:0002752 (“cell surface pattern recognition receptor signaling pathway”), and GO:0002753 (“cytoplasmic pattern recognition receptor signaling pathway”) for pattern recognition receptor genes; GO:0008305 (“integrin complex”), GO:0033627 (“cell adhesion mediated by integrin”), and GO:0005178 (“integrin binding”) for integrin genes; GO:0008237 (“metallopeptidase activity”) for metallopeptidase genes.

#### Cell-cell communication analysis

To explore intercellular communication, CellPhoneDB v2.0 (46) based on ligand and receptor gene expression was employed using the human homologs of DEGs described above. The statistical analysis module was run with 1,000 permutations and a p-value threshold of 0.05. Ligand-receptor pairs were included only if they were expressed in >10% of cells in the interacting IM subsets. Significant pairs were categorized according to known biological functions. The results were subsequently mapped back to mouse genes, and corresponding expression levels were recalculated accordingly.

#### Data visualization

Data visualization involved several tools and packages:

1. R and ggplot2: Multilayer pie chart was generated and a range of plots were modified using the ggplot2 package in R (74). Other plots followed methods detailed in (81–86).
2. Seurat: The Seurat package (76) was utilized for generating FeaturePlot, DimPlot, and VlnPlot visualizations.
3. MAGIC: the MAGIC algorithm (87) was applied to smooth scRNA-seq expression data for the violin plot visualization.
4. SeuratExtend (v.1.1.0): For visualization and statistical comparison of gene expression between different IM subsets, the SeuratExtend package (88), particularly the VlnPlot2 function, was utilized.
5. scCustomize: For visualization of dot plots to include clustering of genes and IM subsets, the scCustomize package (89), particularly the Clustered_DotPlot function, was utilized.
6. ComplexHeatmap: The ComplexHeatmap package (90) was utilized to create heatmaps with customizable annotations.
7. Circlize: The Circlize package (91) was utilized to create chord diagrams for visualizing complex interrelationships among IM subsets and signaling pathways.
8. iTALK: The iTALK package (92) was utilized to create network plots to illustrate interactions among different IM subsets.

## Data Availability

The sequencing raw data and processed scRNA-seq data used in this article are available in the Gene Expression Omnibus (GEO) under accession code GSE225664. The processed scRNA-seq data are also available for online visualization at https://cells.ucsc.edu/?ds=lung-interstitial-macrophage. The Xenium raw data and processed data used in this article are available in the GEO under accession code GSE293144.
