# Supplementary material for: Molecular and spatial specialization of lung interstitial macrophage subsets: beyond chemokines
Source: Front Immunol. 2026 Jun 10;17:1824159. doi: 10.3389/fimmu.2026.1824159 (PMC13290454; doi:10.3389/fimmu.2026.1824159)
Supplement: Supplementary file 1 [file Presentation1.pdf]

Figure S1

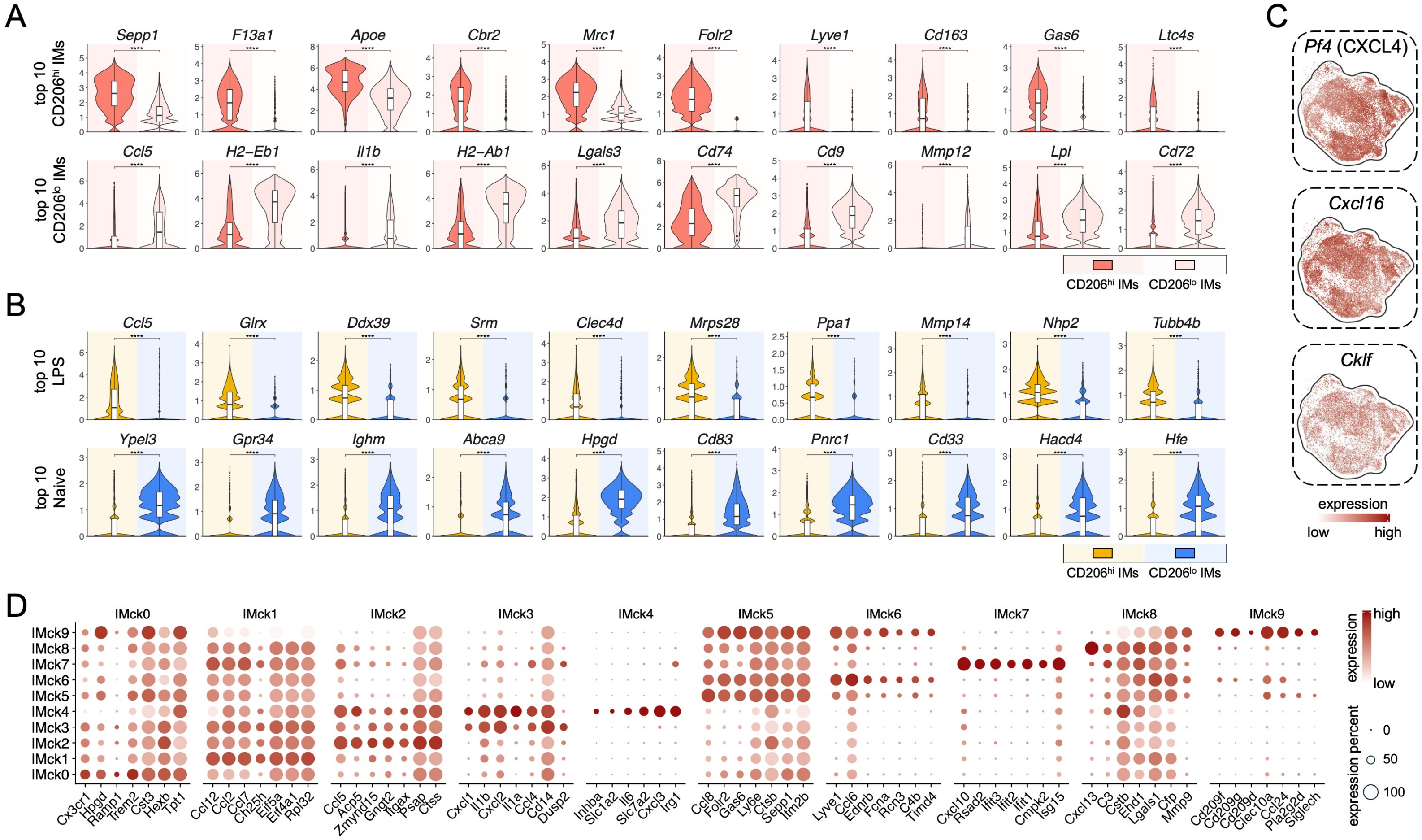

Figure S1. Established IM heterogeneity and coordinated chemokine expression profiles. (**A**) Violin plot comparing the top 10 DEGs in CD206^{hi} IMs and CD206^{lo} IMs. Within each violin, a box plot spans the interquartile range (25th to 75th percentiles) with a horizontal line at the median; whiskers extend to 1.5 × the interquartile range. *P* values calculated using Wilcox test. **P* < 0.05; ***P* < 0.01; ****P* < 0.001; *****P* < 0.0001; nonsignificant results not shown. (**B**) Violin plot comparing the top 10 DEGs in naive IMs and IMs treated with LPS. Within each violin, a box plot spans the interquartile range (25th to 75th percentiles) with a horizontal line at the median; whiskers extend to 1.5 × the interquartile range. *P* values calculated using Wilcox test. **P* < 0.05; ***P* < 0.01; ****P* < 0.001; *****P* < 0.0001; nonsignificant results not shown. (**C**) Feature plots displaying universal expression of *Pf4* (CXCL4), *Cxcl16*, and *Cklf*. (**D**) Dot plot highlighting the top 7 DEGs in each IMck subset.

Figure S2

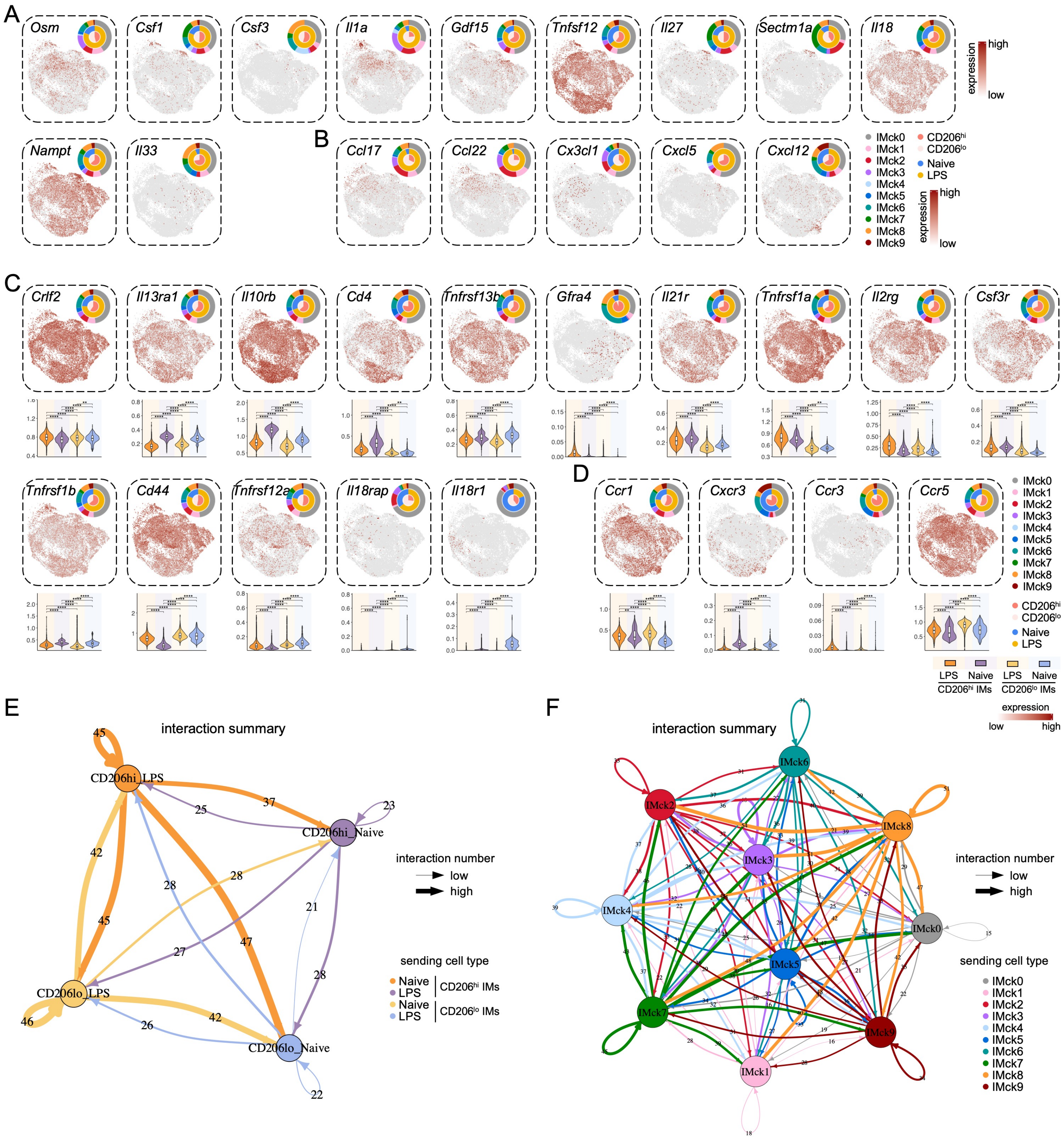
A
Osm
Csf1
Csf3
Il1a
Gdf15
Tnfsf12
Il27
Sectm1a
Il18
Nampt
Il33
expression
high
low
B
Ccl17
Ccl22
Cx3cl1
Cxcl5
Cxcl12
IMck0
IMck1
IMck2
IMck3
IMck4
IMck5
IMck6
IMck7
IMck8
IMck9
CD206hi
CD206lo
Naive
LPS
C
Crlf2
Il13ra1
Il10rb
Cd4
Tnfrsf13b
Gfra4
Il21r
Tnfrsf1a
Il2rg
Csf3r
Tnfrsf1b
Cd44
Tnfrsf12a
Il18rap
Il18r1
D
Ccr1
Cxcr3
Ccr3
Ccr5
LPS
Naive
CD206hi IMs
CD206lo IMs
E
interaction summary
CD206hi_LPS
CD206hi_Naive
CD206lo_LPS
CD206lo_Naive
interaction number
low
high
sending cell type
Naive
LPS
CD206hi IMs
CD206lo IMs
F
interaction summary
interaction number
low
high
sending cell type

Figure S2. Differential expression of cytokine (and chemokine) and receptor genes among IMs. (**A**) Feature plots displaying the differential expression of cytokine genes, with categories annotated as a multilayer pie chart. (**B**) Feature plots displaying the differential expression of chemokine genes, with categories annotated as a multilayer pie chart. (**C**) Feature plots displaying the differential expression of cytokine receptor genes, with categories annotated as a multilayer pie chart, followed by corresponding violin plots comparing these expressions. Within each violin, a box plot spans the interquartile range (25th to 75th percentiles) with a horizontal line at the median; whiskers extend to 1.5 × the interquartile range. *P* values calculated using Wilcox test. **P* < 0.05; ***P* < 0.01; ****P* < 0.001; *****P* < 0.0001; nonsignificant results not shown. (**D**) Feature plots displaying the differential expression of chemokine receptor genes, with categories annotated as a multilayer pie chart, followed by corresponding violin plots comparing these expressions. Within each violin, a box plot spans the interquartile range (25th to 75th percentiles) with a horizontal line at the median; whiskers extend to 1.5 × the interquartile range. *P* values calculated using Wilcox test. **P* < 0.05; ***P* < 0.01; ****P* < 0.001; *****P* < 0.0001; nonsignificant results not shown. (**E**) Network plot representing the sum of significant autocrine interactions across $CD206^{hi}$/$CD206^{lo}$ IMs under different treatments. (**F**) Network plot representing the sum of significant autocrine interactions within different IMck subsets.

Figure S3

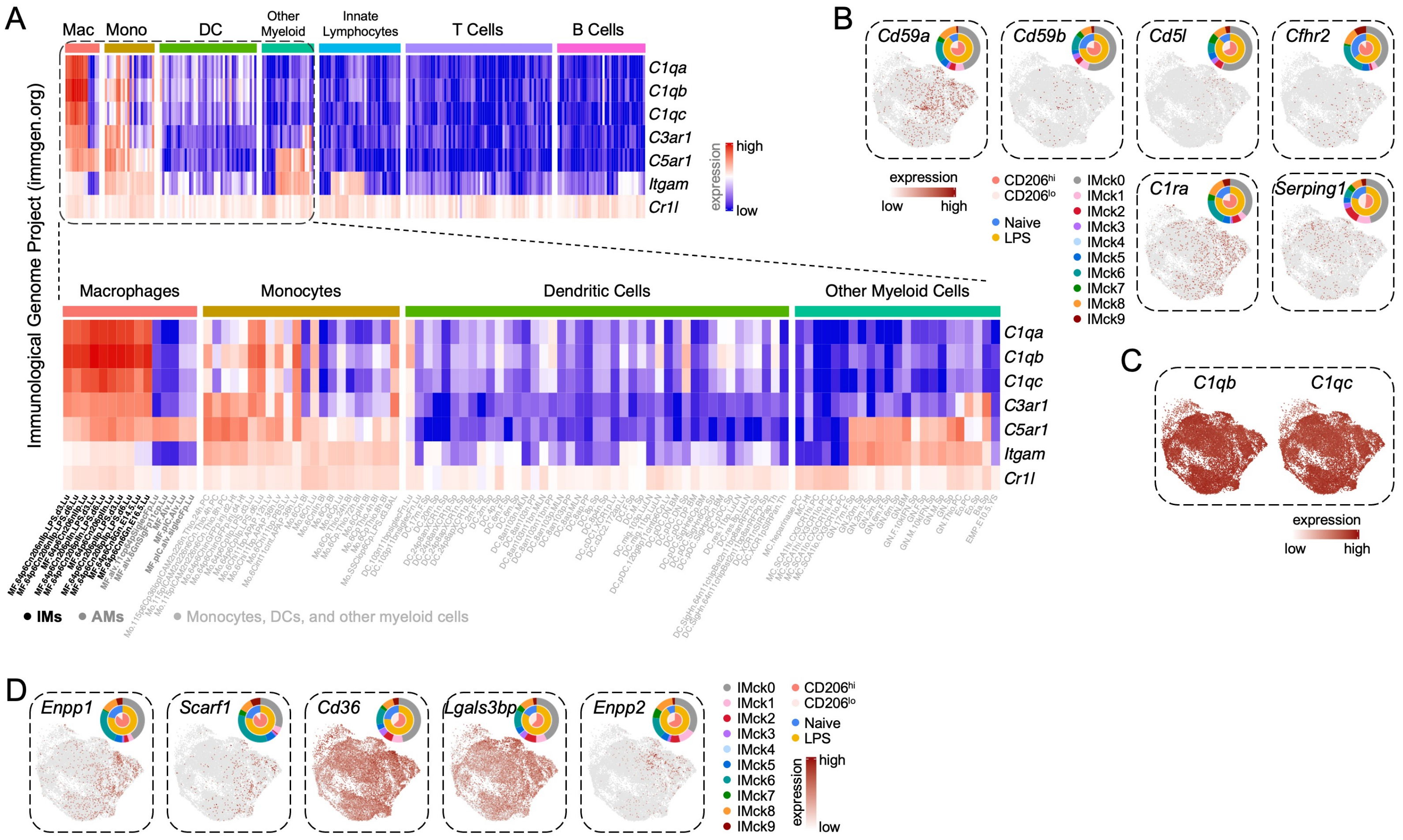

Figure S3. Differential expression of complement system and scavenger receptor genes among IMs. (**A**) Heat maps visualizing the expression of complement system genes universally expressed by IMs in hematopoietic cells based on ImmGen datasets. (**B**) Feature plots displaying the differential expression of complement system genes, with categories annotated as a multilayer pie chart. (**C**) Feature plots displaying the differential expression of *C1qb* and *C1qc*. (**D**) Feature plots displaying the differential expression of scavenger receptor genes, with categories annotated as a multilayer pie chart.

Figure S4

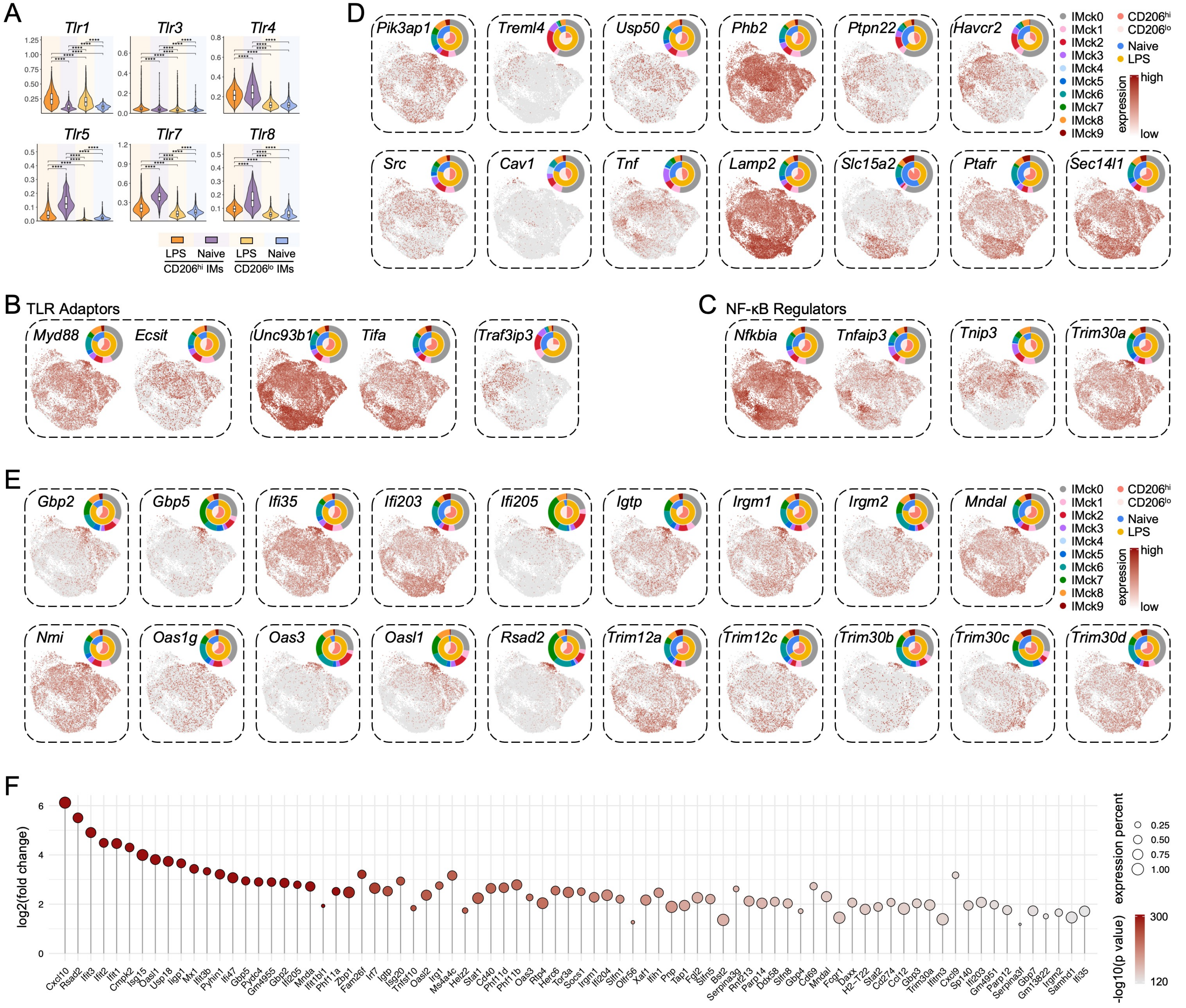

Figure S4. Differential expression of pattern recognition receptor genes among IMs. (**A**) Violin plot comparing the expression of TLR genes across CD206^{hi}/CD206^{lo} IMs under different treatments. Within each violin, a box plot spans the interquartile range (25th to 75th percentiles) with a horizontal line at the median; whiskers extend to 1.5 × the interquartile range. *P* values calculated using Wilcox test. **P* < 0.05; ***P* < 0.01; ****P* < 0.001; *****P* < 0.0001; nonsignificant results not shown. **B**–**C** Feature plots displaying the differential expression of TLR adaptor genes (**B**) and NF-κB regulator genes (**C**), with categories annotated as a multilayer pie chart. (**D**) Feature plots displaying the differential expression of other pattern recognition receptor genes, with categories annotated as a multilayer pie chart. (**E**) Feature plots displaying enriched expression of pattern recognition receptor genes in IMck7, with categories annotated as a multilayer pie chart. (**F**) Lollipop plot representing the top 80 DEGs in IMck7.

Figure S5

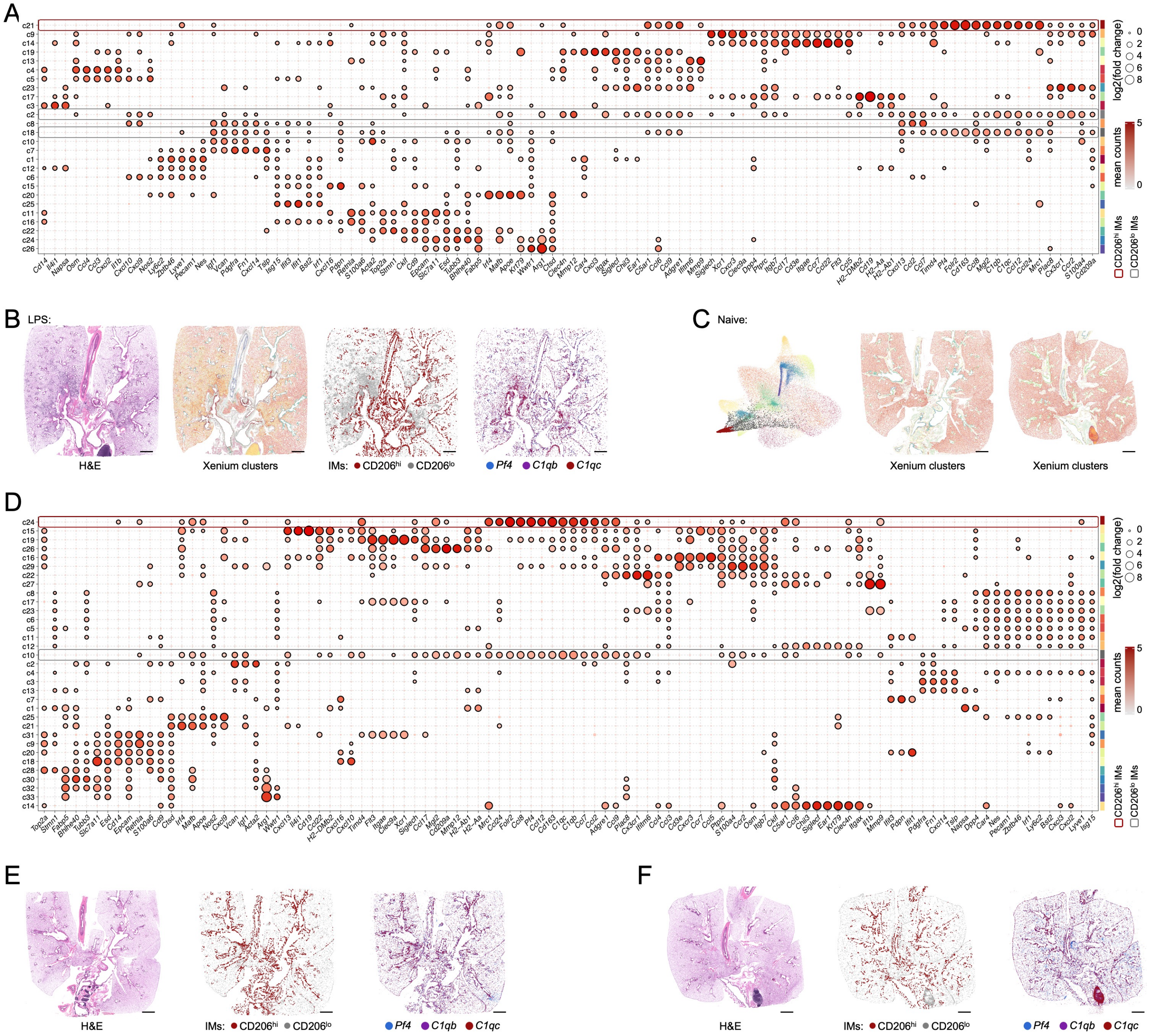

Figure S5. Identification and Spatial Localization of IM Subsets in Xenium Lung Samples. (**A**) Dot plot illustrating expression of integrin and metallopeptidase genes across graph-based cell clusters from LPS-treated Xenium lung samples. Genes and clusters are grouped by similarity, with clusters annotated by color on the right. Boxes highlight CD206^{hi} and CD206^{lo} IM clusters. (**B**) Images from the second LPS-treated lung sample: H&E-stained section, Xenium UMAP plot showing graph-based cell clusters, Xenium spatial localization image highlighting CD206^{hi} and CD206^{lo} IM clusters, and Xenium image illustrating the expression of IM marker genes (*Pf4*, *C1qb*, and *C1qc*) (scale bars, 1,000 μm). (**C**) Xenium UMAP plots and Xenium images displaying graph-based cell clustering of the two naive lung samples. (**D**) Dot plot illustrating expression of integrin and metallopeptidase genes across graph-based cell clusters from naive Xenium lung samples. Genes and clusters are grouped by similarity, with clusters annotated by color on the right. Boxes highlight CD206^{hi} and CD206^{lo} IM clusters. **E–F.** Images from the two naive lung samples, each including an H&E-stained section, Xenium spatial localization image highlighting CD206^{hi} and CD206^{lo} IM clusters, and Xenium image illustrating the expression of IM marker genes (*Pf4*, *C1qb*, and *C1qc*) (scale bars, 1,000 μm).

Figure S6

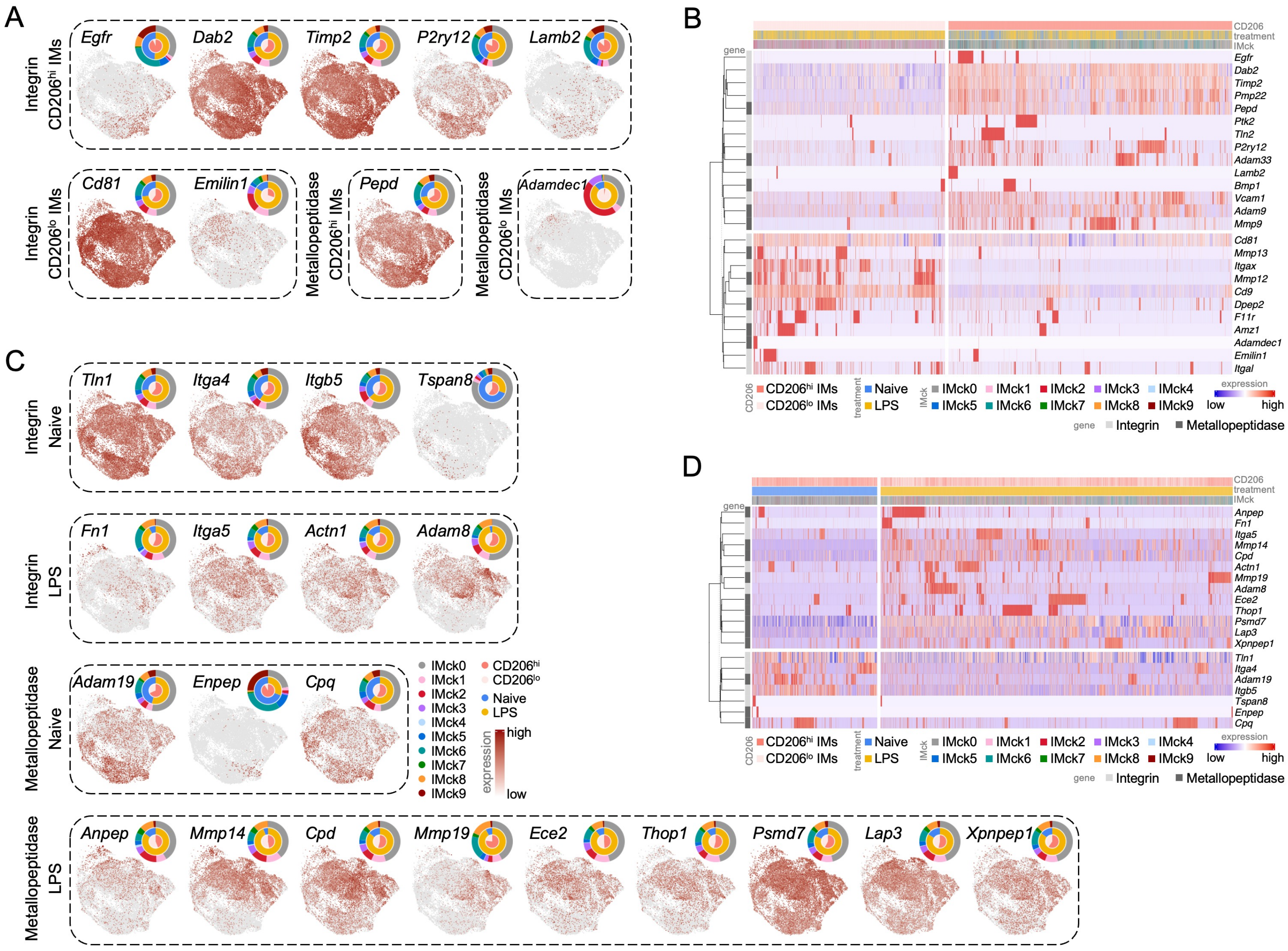

Figure S6. Differential expression of integrin and metallopeptidase genes among IMs. (**A**) Feature plots displaying the differential expression of integrin and metallopeptidase genes within CD206^{hi}/CD206^{lo} IMs, with categories annotated as a multilayer pie chart. Genes are shown by different classes. (**B**) Heat map visualizing integrin and metallopeptidase gene expression in individual CD206^{hi}/CD206^{lo} IMs, with categories annotated on top. Genes are annotated by class on the left. (**C**) Feature plots displaying the differential expression of integrin and metallopeptidase genes within naive IMs and IMs treated with LPS, with categories annotated as a multilayer pie chart. Genes are shown by different classes. (**D**) Heat map visualizing integrin and metallopeptidase gene expression in naive IMs and IMs treated with LPS, with categories annotated on top. Genes are annotated by class on the left.

Figure S7

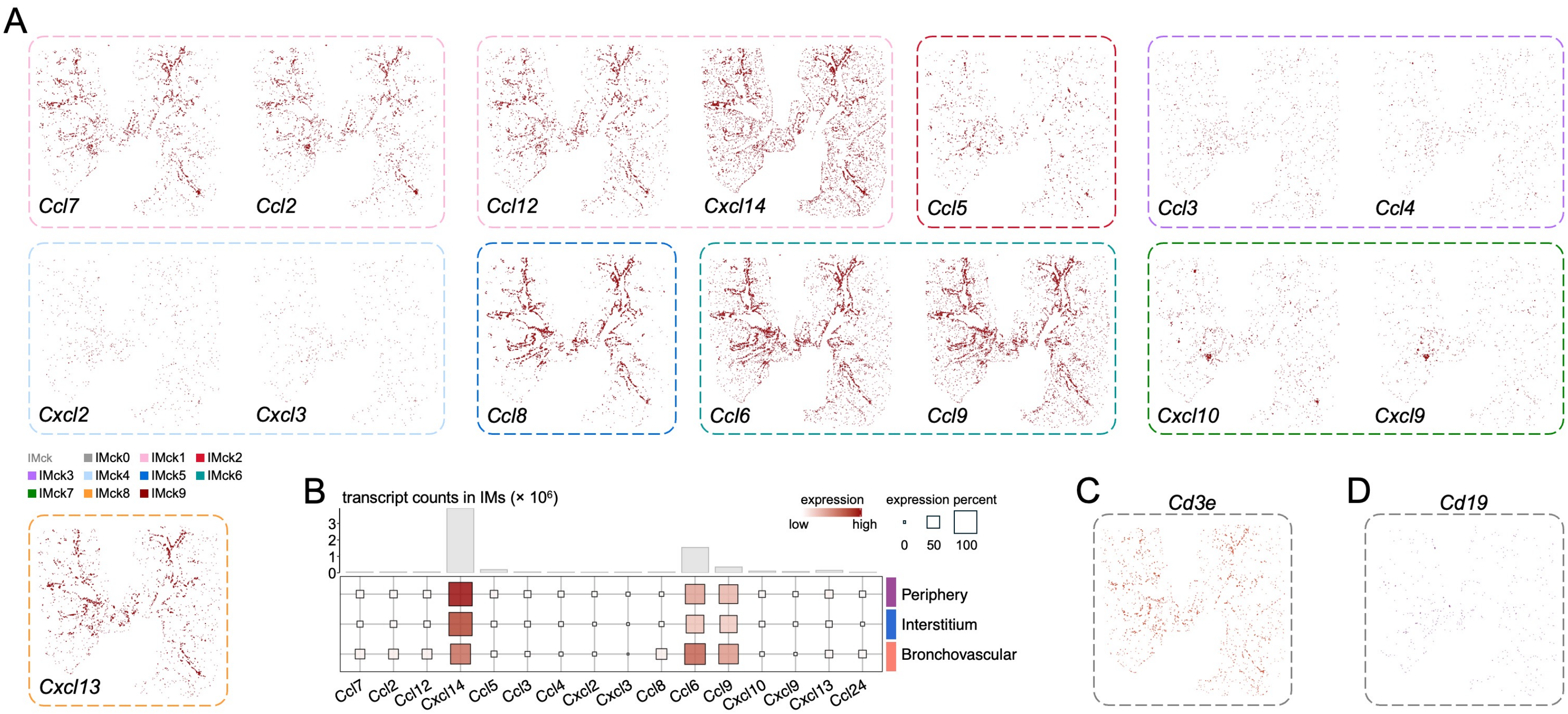

Figure S7. Differential localization of chemokine-expressing IMck subsets in naive lung. (**A**) Representative Xenium image showing chemokine gene expression in naive lung IMs. Chemokine expression combinations defining IMck subsets are indicated by colored dotted boxes corresponding to each IMck subset. *Ccl24* is shown in **Figure 5C**. (**B**) Dot plot highlighting differential localization patterns and total transcript counts of chemokine expression within naive lung IMs. **C**–**D** Representative Xenium image showing *Cd3e* and *Cd19* expression in the naive lung.

Figure S8

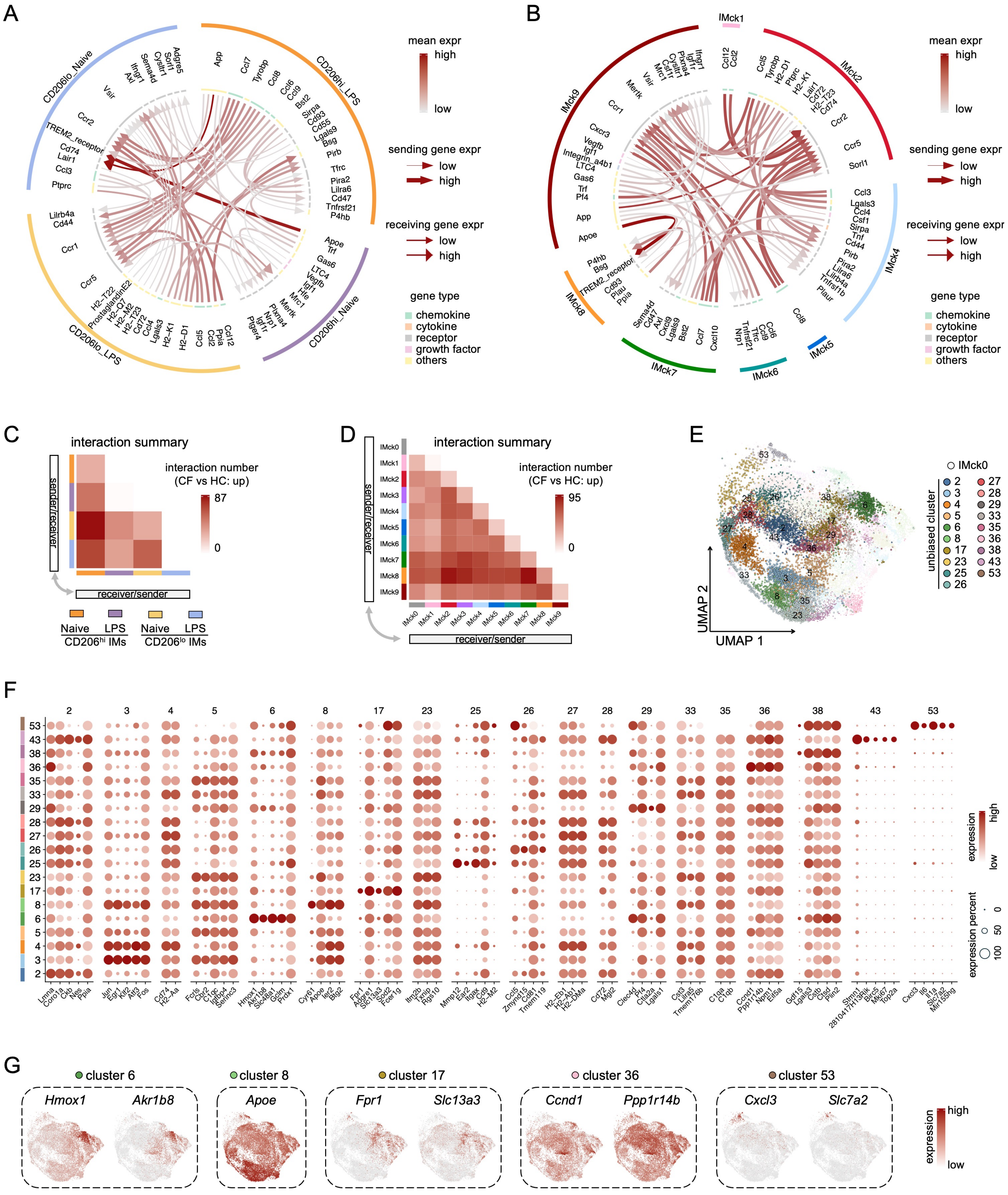
A
B
C
D
E
F
G
mean expr
high
low
sending gene expr
receiving gene expr
gene type
chemokine
cytokine
receptor
growth factor
others
CD206lo_Naive
CD206hi_LPS
CD206hi_Naive
CD206lo_LPS
IMck1
IMck2
IMck4
IMck5
IMck6
IMck7
IMck8
IMck9
interaction summary
interaction number
(CF vs HC: up)
87
95
0
sender/receiver
receiver/sender
Naive
LPS
CD206hi IMs
CD206lo IMs
IMck0
UMAP 1
UMAP 2
unbiased cluster
expression
expression percent
cluster 6
cluster 8
cluster 17
cluster 36
cluster 53
Hmox1
Akr1b8
Apoe
Fpr1
Slc13a3
Ccnd1
Ppp1r14b
Cxcl3
Slc7a2

Figure S8. IMs interact with IMs in various ways and the heterogeneity of IMck0. (**A**) Circos plot depicting the top 50 interactions, including all interaction types, where molecules work in an autocrine manner to direct function of $CD206^{hi}$/$CD206^{lo}$ IMs under different treatments. Each segment of the outer circle represents a distinct IM subtype. (**B**) Circos plot depicting the top 50 interactions, including all interaction types, where molecules work in an autocrine manner to direct function of each IMck subset. Each segment of the outer circle represents a distinct IMck subset. Each segment of the inner circle represents different genes, color-coded by molecule type. Arcs indicate ligand-receptor interactions, with line thickness proportional to ligand gene expression and arrow width proportional to receptor gene expression. Arcs are color-coded by mean expression value. (**C**) Heat map visualizing the sum of significant autocrine interactions across $CD206^{hi}$/$CD206^{lo}$ IMs under different treatments. (**D**) Heat map visualizing the sum of significant autocrine interactions within different IMck subsets. (**E**) UMAp plots illustrating the high-resolution, unbiased clustering analysis of IMck0. Unstroked triangular markers in different colors represent unbiased clusters within other IMck subsets, while stroked dots in different colors represent unbiased clusters specifically identified within IMck0. (**F**) Dot plot highlighting the top 5 DEGs in each unbiased cluster within IMck0. (**G**) Feature plots displaying the differential expression of DEGs from several unbiased clusters within IMck0.
